# A novel small molecule ZYZ384 targeting SMYD3 for hepatocellular carcinoma via reducing H3K4 trimethylation of the Rac1 promoter

**DOI:** 10.1002/mco2.711

**Published:** 2024-09-15

**Authors:** Qian Ding, Jianghong Cai, Li Jin, Wei Hu, Wu Song, Peter Rose, Zhiyuan Tang, Yangyang Zhan, Leilei Bao, Wei Lei, Yi Zhun Zhu

**Affiliations:** ^1^ State Key Laboratory of Quality Research in Chinese Medicine & Laboratory of Drug Discovery from Natural Resources and Industrialization & School of Pharmacy Macau University of Science and Technology Macau SAR China; ^2^ Affiliated Hospital of Guangdong Medical University Zhanjiang China; ^3^ Joint Laboratory of TCM Innovation (Transformation) of Guizhou and Macau Guizhou University of Traditional Chinese Medicine Guiyang China; ^4^ School of Biosciences University of Nottingham Loughborough UK; ^5^ Department of Pharmacy Affiliated Hospital of Nantong University & Medical School of Nantong University Nantong China; ^6^ Department of Pharmacy, Shanghai Eastern Hepatobiliary Surgery Hospital Navy Military Medical University Shanghai China; ^7^ Shanghai Key Laboratory of Bioactive Small Molecules, Department of Pharmacology, School of Pharmacy Fudan University Shanghai China

**Keywords:** antitumor, H3K4me3, Rac1 promoter, small molecule, SMYD3, ZYZ384

## Abstract

SMYD3 (SET and MYND domain‐containing 3) is a histone lysine methyltransferase highly expressed in different types of cancer(s) and is a promising epigenetic target for developing novel antitumor therapeutics. No selective inhibitors for this protein have been developed for cancer treatment. Therefore, the current study describes developing and characterizing a novel small molecule ZYZ384 screened and synthesized based on SMYD3 structure. Virtual screening was initially used to identify a lead compound and followed up by modification to get the novel molecules. Several technologies were used to facilitate compound screening about these novel molecules' binding affinities and inhibition activities with SMYD3 protein; the antitumor activity has been assessed in vitro using various cancer cell lines. In addition, a tumor‐bearing nude mice model was established, and the activity of the selected molecule was determined in vivo. Both RNA‐seq and chip‐seq were performed to explore the antitumor mechanism. This work identified a novel small molecule ZYZ384 targeting SMYD3 with antitumor activity and impaired hepatocellular carcinoma tumor growth by reducing H3K4 trimethylation of the Rac1 promoter triggering the tumor cell cycle arrest through the AKT pathway.

## Graphical abstract

The current study describes developing and characterizing a novel small molecule ZYZ384 screened and synthesized based on SMYD3 structure. ZYZ384 targeted SMYD3 with antitumor activity and impaired hepatocellular carcinoma tumor growth by reducing H3K4 trimethylation of the Rac1 promoter triggering the tumor cell cycle arrest through the AKT pathway.

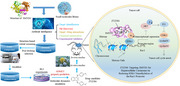

## 1 INTRODUCTION

Cancer(s) are still the leading cause of disease‐related death worldwide, and epigenetic systems have become important targets for antitumor research.^1^, ^2^ Epigenetics is the study of heritable changes in gene expression that occur without alterations to the underlying DNA sequence. However, these modifications are mainly reversible. The epigenetic modification mechanisms include DNA methylation, chromatin remodeling, histone modifications, and RNA interference.^3^ These post‐translational changes are intimately linked to tumorigenesis and cellular development.

In recent times, it has been reported that the histone methyltransferase SMYD3 (SET and MYND domain‐containing protein 3) is highly expressed in various human tumors and that overexpression enhances K‐Ras‐associated signaling in cancer cells.^4^, ^5^ To date, SMYD3 overexpression in cancerous tissues has been reported to occur in liver cancer, colon cancer,^6^ breast cancer,^7^ pancreatic cancer,^8^ prostate cancer,^9^ bladder cancer,^10^ gastric cancer,^11^ malignant glioma,^12^ esophageal squamous cell carcinoma,^13^ chronic lymph cell leukemia,^14^ and cervical cancer.^15^ This information points to SMYD3 as a cancer drug discovery and development candidate. At present, there are few descriptions of molecules that can inhibit SMYD3 in the literature: several have issues linked to low inhibition efficiency,^16^ or are not applied to in vivo study.^17^ Although there is a small molecule SMYD3 inhibitor that is considered to have good bioavailability,^18^ or others can inhibit cancer cell proliferation,^19^, ^20^ currently, no molecules targeting SMYD3 have been reported to be used in clinical cancer treatment.

Our current discovery of the novel small molecule targeting SMYD3 may open a new avenue in future cancer treatment.

Opportunities for using technologies by researchers in drug discovery are becoming more widely available and facilitating the identification of novel drug molecules. Indeed, computer‐assisted drug virtual screening models have allowed for the assessment of drug screening through computer simulation technology and can be used in the evaluation of docking small molecules to protein targets.^21^ Similarly, biolayer interferometry (BLI), a label‐free technology for detecting biomolecular interactions, can provide high‐throughput biomolecular interaction information in real time and offers kinetic and affinity information. Studies assessing the interaction between inhibitors and proteins can often be challenging, so molecular dynamics (MD) simulations can be helpful in evaluating the stable binding pattern of ligands to proteins. Moreover, MD simulations enable us to derive trajectories containing all information about the stability and relationships of molecular interactions between ligand–protein complexes.^22^

In the current research, we have characterized drug‐like molecules initially identified via a virtual screening of small molecular weight compound libraries. This research led to the identification of a series of lead compounds that were assessed based on their inhibitory potential of SMYD3 (please refer to the Supporting Information for details). The lead compound was then used to design and synthesize a series of novel small molecules. These molecules were tested in vitro and in vivo models for SMYD3 inhibitory properties and anticancer activities.

## 2 RESULTS

### 2.1 Design and synthesis of novel small molecules targeting SMYD3

In order to get novel molecules targeting SMYD3, we first conducted virtual screening (Figure S1) to identify lead compound 1 (Table S1) and modified its structure. Two series of novel small molecules were obtained from the modification and synthesis of lead compound 1 (Figure 1). For synthesis details please refer to Section 4. Nuclear magnetic resonance (NMR) verified the structure of each compound. The NMR and high‐resolution mass spectrometry (HRMS) of each novel small molecule, please refer to the Figures S2–S37 for details. Two series of novel small molecules were successfully synthesized for subsequent experiments.

**FIGURE 1:**
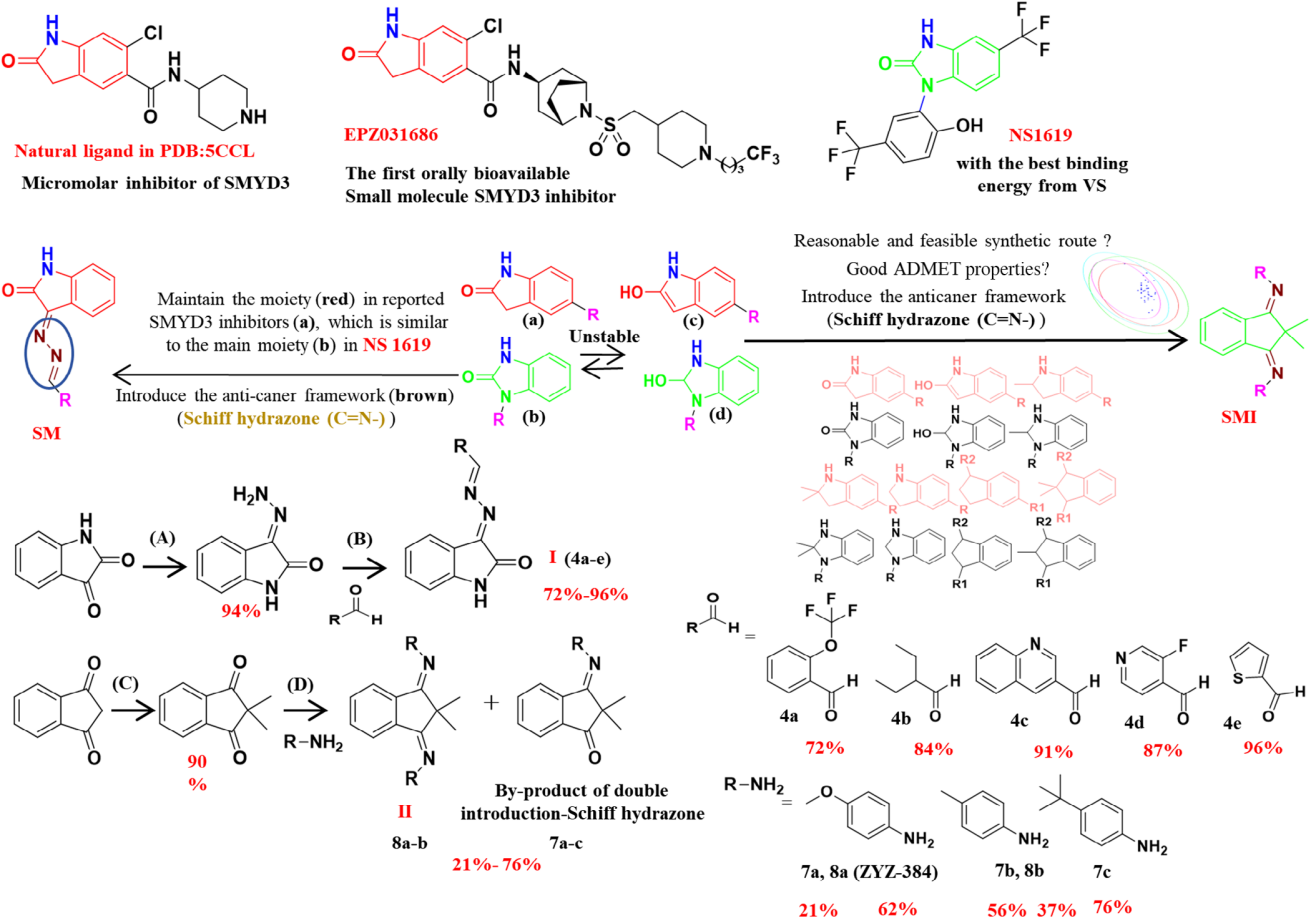
Design and synthesis of novel small molecules targeting SMYD3. Reagents and conditions: (A) NH_2_NH_2_·H_2_O, methanol, reflux, 3 h; (B) AcOH, methanol, reflux, 8 h; (C) CH_3_I, potassium fluoride, acetonitrile, 70°C, 24 h; (D) *p*‐toluenesulfonic acid, toluene, reflux, 8 h.

### 2.2 ZYZ384 (SMI1) has a good binding affinity to SMYD3

Next, to test the binding affinity of novel small molecules to SMYD3, BLI analyses were performed to determine the binding affinity of the two series of novel small molecules targeting SMYD3. SMYD3‐glutathione S‐transferase (GST)‐tagged protein was immobilized onto GST biosensors and used to determine the binding affinity of all respective small molecules and labeled probes. The binding and dissociation curves were generated in real time by applying the SMYD3‐GST label proteins onto the biosensor surfaces, followed by monitoring small molecules interaction across several concentrations. The results for the SM and SMI series of compounds are shown in Table 1. SMI1 shows the good binding affinity of all compounds tested (*R* ^2^ > 0.95, *K*_D_ < 10 E‐05). The binding curves for this interaction suggest a simple 1:1 binding pattern (*R* ^2^ = 0.953), the equilibrium dissociation constant (*K*_D_) of the interaction between SMYD3 and SMI1 was determined to be 78 µM (steady‐state analysis, lower panel), better than two commercial inhibitors EPZ031686 (*K*_D_: 195 µM) and BCI‐121 (*K*_D_: 114 µM). These results show that SMI1 interacts and binds to SMYD3 protein and has a good binding affinity to SMYD3. SMI1 has been renamed ZYZ384 and further tested for anticancer activities using both in vitro and in vivo models.

**TABLE 1:** The binding affinity of small molecules to the target protein SMYD3.

Compd	*R* ^2^	*K*_D_ (M)
SMI1/8a	0.953	7.80 E‐05
SMI3/8b	0.7514	4.47E‐05
SMI11/7a	0.9085	1.15 E‐04
SMI12/7c	0.5939	6.99 E‐06
SMI13/7b	0.7926	9.57 E‐05
SM20/4a	0.933	2.16E‐05
SM17/4b	0.4431	4.67E‐06
SM28/4c	0.8129	2.76E‐06
SM31/4d	0.8931	2.60E‐04
SM23/4e	0.9218	2.10E‐05
EPZ031686	0.9745	1.95E‐04
BCI‐121	0.9493	1.14E‐04

### 2.3 ZYZ384 binding with SMYD3 and inhibits SMYD3 activity

**FIGURE 2:**
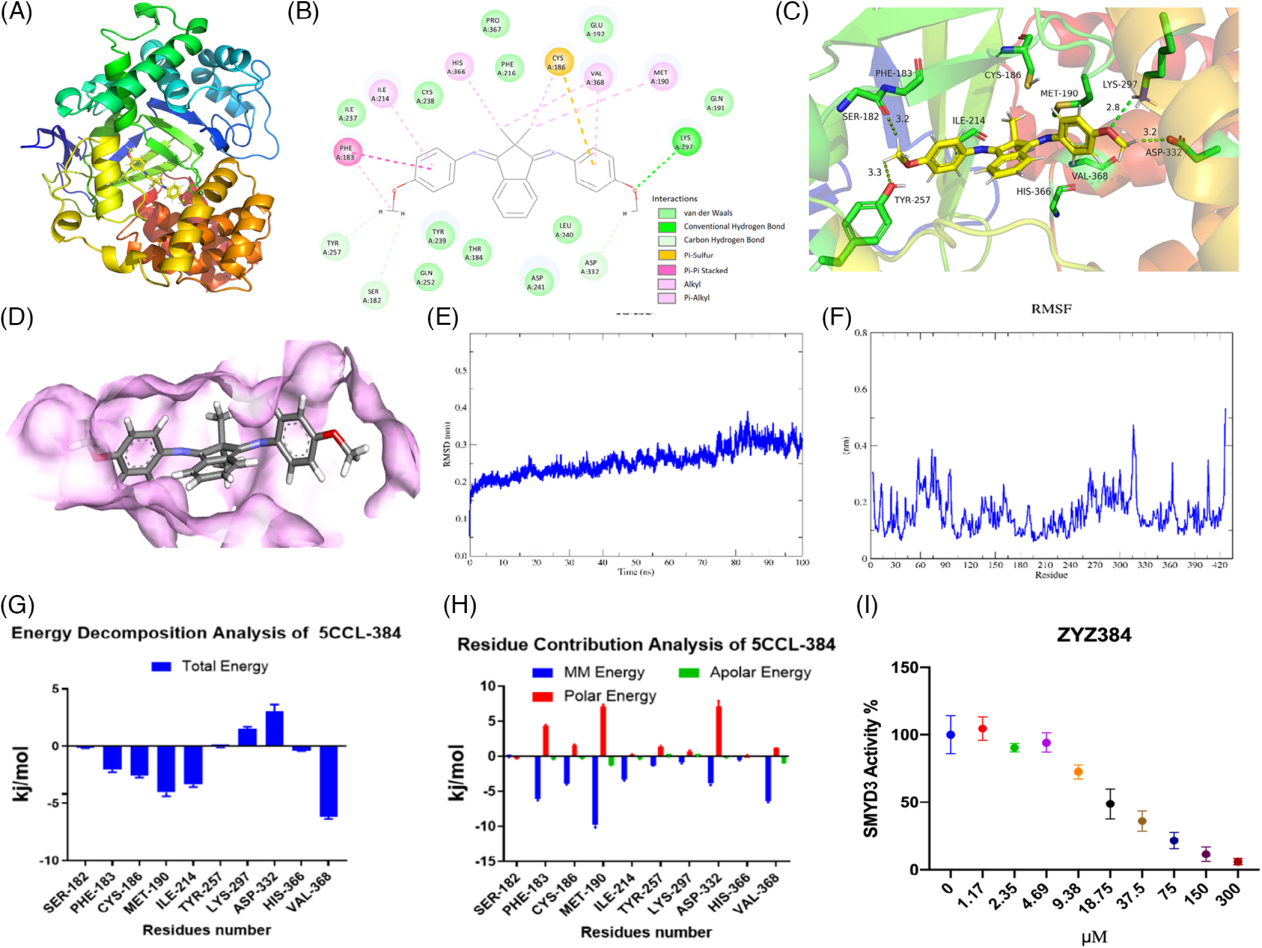
ZYZ384 binds and inhibits SMYD3 activity. (A) The best combination mode; (B) the two‐dimensional (2D) interaction of ZYZ384 and SMYD3); (C) three‐dimensional interaction of ZYZ384 and SMYD3; (D) the active binding pocket; (E) the root mean square displacement during the molecular dynamics (MD) simulation; (F) the root mean square fluctuation during the MD simulation; (G) the energetic decomposition of main individual residues; (H) the energetic contribution of main individual residues; (I) the SMYD3 Homogeneous Assay Kit was used to detect the SMYD3 activity exposed to different concentrations of ZYZ384. The alpha counts were recorded and analyzed with GraphPad 8.0.

The previous data show that ZYZ384 and SMYD3 have good binding affinity. Next, we used computer simulation methods to predict possible binding sites and modes. Molecular docking was assessed to predict the binding site interaction of ZYZ384 with SMYD3 protein. The binding energy of ZYZ384 and SMYD3 was calculated to be −8.73 kcal/mol with a reasonable combining pocket; this pocket contributes to maintaining a stable conformation of ZYZ384 and SMYD3 (Figure 2A: Best combination mode; D: Active binding pocket). The binding region includes multiple amino acid residues, namely, SER‐182, PHE‐183, CYS‐186, MET‐190, ILE‐214, TYR‐257, LYS‐297, ASP‐332, HIS‐366, and VAL‐368 (Figure 2B: two‐dimensional [2D] interaction of ZYZ384 and SMYD3). The interaction included the formation of hydrogen bonds, van der Waals forces, Pi–Pi conjugation, and other interactions. Indeed, the methoxy group on ZYZ384 forms hydrogen bond interactions with TYR‐257, SER‐182, ASP‐332, and LYS‐297 of SMYD3 protein with an average hydrogen bond distance of 3.125 Å. PHE‐183, ILE‐214, HIS‐366, VAL‐368, and MET‐190 of SMYD3 (Figure 2C: three‐dimensional [3D] interaction of ZYZ384 and SMYD3). The reported interactions are in maintaining the stability of the ZYZ384 and SMYD3 complexes.

The root mean square displacement (RMSD) was used to measure the average distance between atoms and reveal any structural changes between SMYD3 and target compounds over time. High RMSD values indicate the target protein undergoing structural changes via conformational rearrangement induced by the ligand to fit binding pockets. In contrast, lower deviations indicate complex stability. According to the RMSD analyses, the complex system trajectories are comparatively stable from 0 ns, with RMSD values fluctuating within 0.1 nm (Figure 2E). This result indicates that the systems had reached convergence. Toward the end of the simulation (80‐100 ns), the complex showed a low RMSD (about 0.3 nm) with a smaller conformational change. This indicates a higher interaction efficiency and good stability of the ZYZ384, SMYD3 interaction.

The root mean square fluctuation (RMSF) was calculated from the movement of each residue along the trajectory around the mean position, revealing the flexibility of specific protein regions during MD simulations. The larger RMSF values correspond to regions with greater freedom that is potentially located in the active site at residues SER‐182, PHE‐183, CYS‐186, MET‐190, ILE‐214, TYR‐257, LYS‐297, HIS‐366, and VAL‐368. All values for RMSF were consistent with the molecular docking results (Figure 2F). The residues above 400 have the largest fluctuation, and these residues are known to have special functional correlations with RMSF values of more than 0.4 nm.

The binding free energies of ZYZ384 and SMYD3 were calculated to be −94.405 ± 9.50 kcal/mol, respectively. These free energies are representative of van der Waals energy (−173.890 ± 9.798 kcal/mol), electrostatic energy (−13.585 ± 6.080 kcal/mol), polar solvation energy (113.27 ± 10.691 kcal/mol), and Solvent Accessible Surface Area (SASA) nonpolar solvation energy (−20.200 ± 1.042 kcal/mol). We subsequently calculated the energetic contribution of the potential key residues involved in the interactions of ZYZ384 and SMYD3. The results indicated that the following residues are important for the inhibition of SMYD3: SER‐182 (−0.1828 kcal/mol), PHE‐183 (−0.1828 kcal/mol), CYS‐186 (−2.5796 kcal/mol), MET‐190 (−4.0405 kcal/mol), ILE‐214 (−3.3782 kcal/mol), TYR‐257 (−0.0391 kcal/Mol), LYS‐297 (1.5286 kcal/mol), ASP‐332 (3.0213 kcal/mol), HIS‐366 (−0.4137 kcal/mol), and VAL‐368 (−6.1474 kcal/mol). VAL‐368 contributed to the total binding free energies among the identified residues. Interactions with LYS‐297 and ASP‐332 seem unfavorable in this model (Figure 2G). In addition, the energy decomposition of potential thermal residues was determined to identify which interactions dominate the binding free energy (Figure 2H). Discrete energy terms that contribute to binding free energy indicate that Molecular Mechanics (MM) energy (van der Waal energy and electrostatic energy) is the driving component of the binding in the docking complex. Polar solvation energy did not contribute favorably to the total interaction. Finally, the contribution of the nonpolar solvation energy of SASA to binding free energy was similar. The decomposition energy per residue of surrounding moieties indicates that the main beneficial energy contribution of ZYZ384 binding comes from residues PHE‐183, CYS‐186, MET‐190, and VAL‐368. These appear to be critical sites for ZYZ384 and would be important to produce any potential physiological effects following the interaction of this molecule with SMYD3.

The homogeneous assay kit was used to evaluate ZYZ384 inhibition activity on SMYD3. As we know, SMYD3 can catalyze the di‐ and tri‐methylation status of lysine4 of histone H3. This protein is also reported to drive the methylation of a lysine residue present in the protein MAPK/ERK Kinase 2 (MAP3K2). Therefore, a series of different concentrations of ZYZ384 were incubated with SMYD3, and the activity was determined using the kit. The alpha counts were calculated and analyzed and showed that ZYZ384 inhibits SMYD3 in a concentration‐dependent manner.

The above data revealed ZYZ384 inhibits SMYD3 activity and predict the possible binding site and mode with SMYD3.

### 2.4 ZYZ384 inhibits different cancer cell proliferation and suppresses the overexpression of SMYD3

**FIGURE 3:**
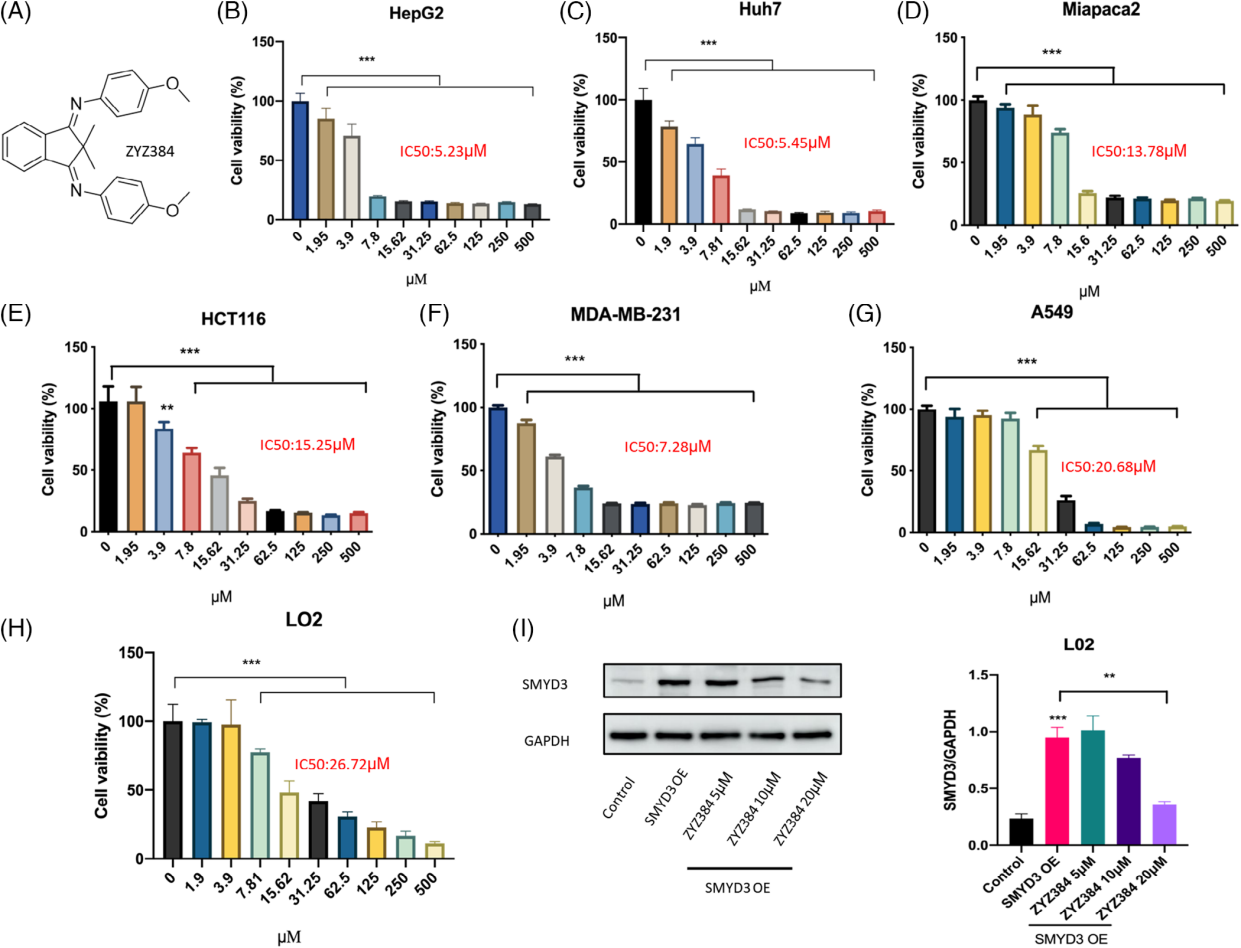
The cytotoxicity of ZYZ384 on different cell lines. (A) The structure of ZYZ384, (B) human liver cancer cell HepG2, (C) human liver cancer cell Huh7, (D) human normal liver cells LO2, (E) human pancreas adenocarcinoma Miapaca2, (F) human colon cancer cell HTC116, (G) human breast cancer cell MDA‐MB‐231, (H) human non–small‐cell lung cancer cell A549, (I) SMYD3 overexpression and ZYZ384 treatment on LO2. Data were expressed as mean ± SEM. ***: *p* < 0.001; **: *p* < 0.01 versus control, one‐way ANOVA analysis.

The cytotoxicity of ZYZ384 was determined across several different cancer cell lines. 3‐(4,5‐Dimethylthiazol‐2‐yl)‐2,5‐diphenyltetrazolium bromide (MTT) assay was used to assess cell viability. The results showed that ZYZ384 (Figure 3A) significantly decreased cancer cell viability in a concentration‐dependent manner after 24 h incubation (Figure 3B–G). The cancer cell lines tested include human liver carcinoma cell HepG2, adenocarcinoma human alveolar basal epithelial cell A549, human colon carcinoma cell HTC116, human breast adenocarcinoma cell MDA‐MB‐231, and human pancreatic adenocarcinoma cell Miapaca2. ZYZ384 demonstrated the most effective inhibitory activity in human hepatoma HepG2 cells and could reduce cell proliferation at low concentrations, namely, IC_50_ = 5.23 µM. In addition, ZYZ384 had low toxicity when tested in normal liver cells (LO2; Figure 3H). SMYD3 overexpression was conducted on LO2 cells by transfection and subsequently suppressed by ZYZ384 (Figure 3I). These results indicated that ZYZ384 Inhibits different cancer cell proliferation and suppress the overexpression of SMYD3.

### 2.5 ZYZ384 is safe with potential druggability

**FIGURE 4:**
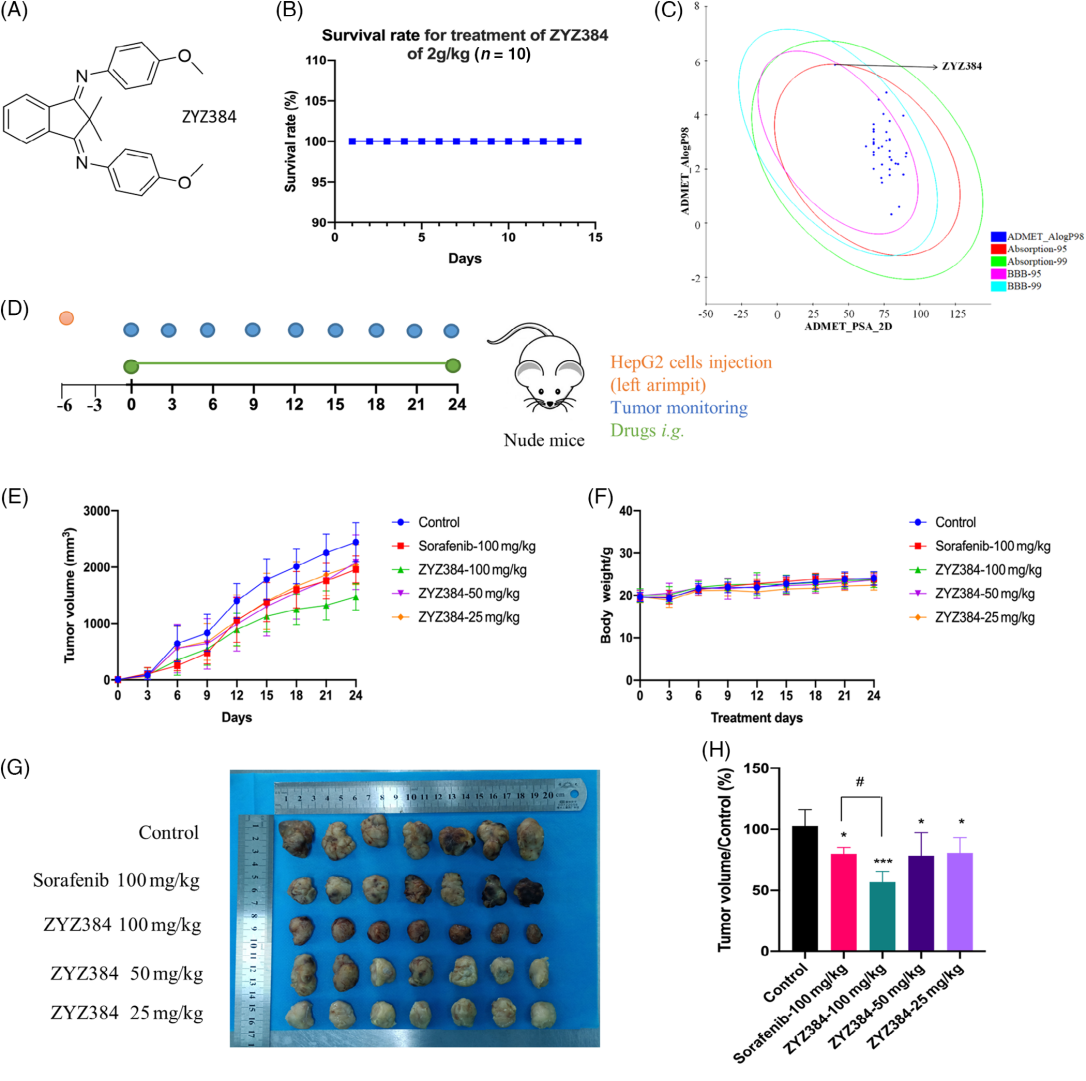
In vivo study and druggability prediction of ZYZ384. (A) The structure of ZYZ384. (B) Safety and attempted evaluation of lethal dose for ZYZ384. C57BL/6 mice were orally administrated with 2 g/kg (*n* = 10) of ZYZ384 for 14 days, and the survival of the mice was monitored and recorded. (C) ADMET analysis of novel small molecules. Discovery Studio software (2020) was used to calculate ADMET properties. A, Absorption; D, Distribution; M, Metabolism; E, Excretion; T, Toxicity, the 99% and 95% confidence intervals of the blood–brain barrier permeability (BBB) model, and the 99% and 95% confidence intervals of the human intestinal absorption (HIA) model. (D) The establishment of tumor‐bearing nude mice model and treatment method. (E) Tumor volume monitoring during treatment. (F) Body weight monitoring during treatment. (G) Tumor volume of each group. (H) Comparison of tumor volume in each group. Data were expressed as mean ± SEM. ADMET, Absorption, Distribution, Metabolism, Excretion, Toxicity. #: *p* < 0.05 versus positive control; ***: *p* < 0.001; *: *p* < 0.05 versus control, one‐way ANOVA analysis.

The toxicity of ZYZ384 (Figure 4A) was determined using C57BL/6 mice in pilot studies to assess the safety and the maximum‐tolerable dose (MTD) determined (as shown in Figure 4B). No toxicity was reported in C57BL/6 mice orally administrated with 2 g/kg of ZYZ384 one time, and a survival rate of 100% was reached after 2 weeks of treatment. No abnormalities in food and water consumption are reported during this time, nor any unusual spontaneous activities; a reported LD5 for ZYZ384 >2 g/kg is noted for the current work. As shown in Figure 4C, the Absorption, Distribution, Metabolism, Excretion, Toxicity (ADMET) plot is a 2D plot of ADMET_PSA_2D versus ADMET_AlogP98. This diagram shows 95% and 99% confidence intervals for the BBB model and 95% and 99% confidence intervals for the HIA model. As indicated, ZYZ384 has salient ADMET properties and shows potential druggability (Figure 4C).

### 2.6 Growth suppression in HepG2 tumors of nude mice by ZYZ384

The in vivo antitumor effect of ZYZ384 was assessed using a HepG2 tumor‐bearing nude mouse model (cell‐derived xenograft, CDX; Figure 4D). The results show that the 100 mg/kg ZYZ384 promotes growth suppression in HepG2 tumors of nude mice that was better than that of sorafenib at 100 mg/kg (Figure 4E,G,H). Moreover, no noticeable body weight changes were observed during treatment (Figure 4F). Taken together, these results show that ZYZ384 is effective at inhibiting the growth of liver tumors in the elected animal model.

### 2.7 ZYZ384 reduced the H3K4me3 occupancy at the transcription start site of the Rac1 gene in tumor tissues

**FIGURE 5:**
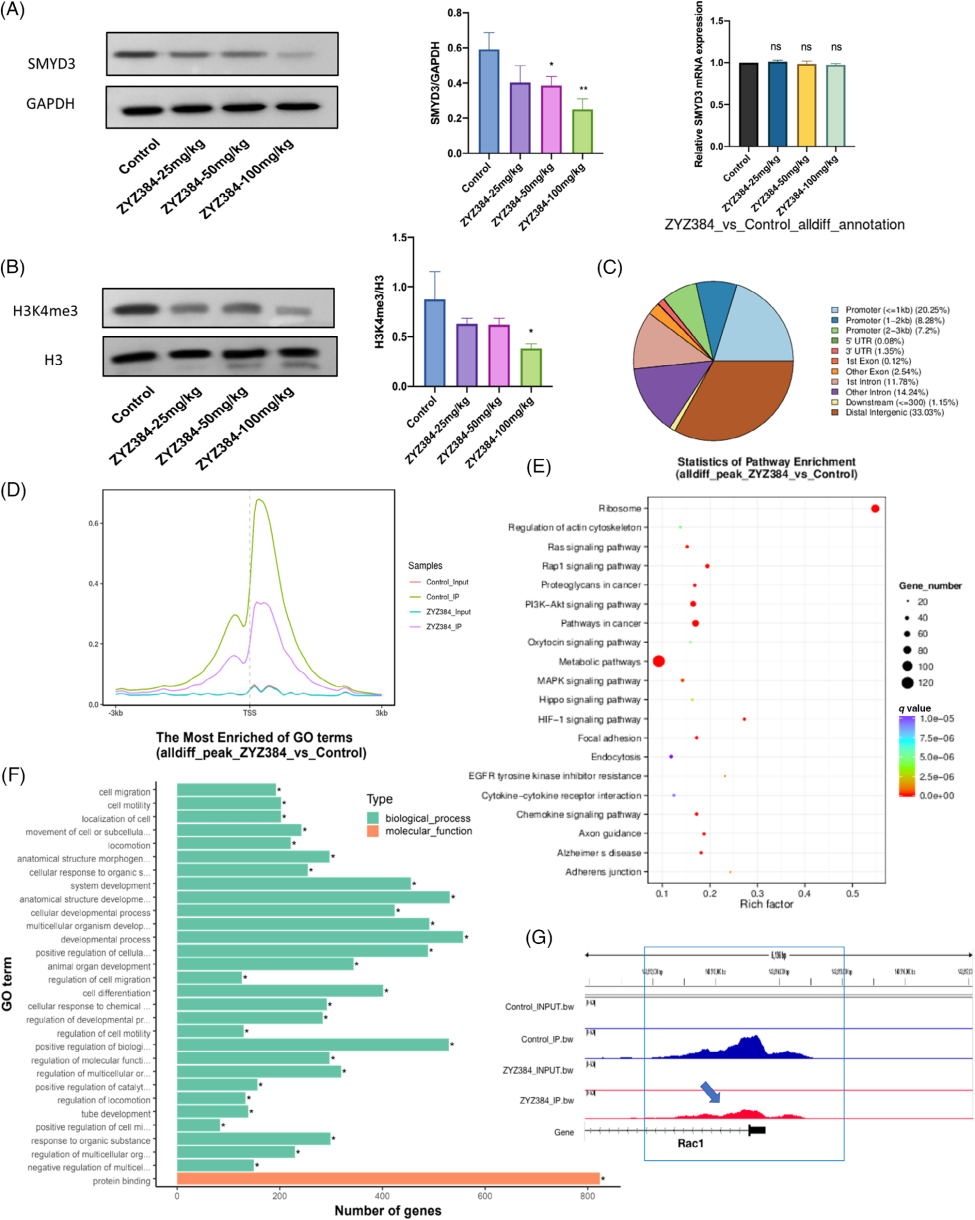
ZYZ384 inhibition effect on SMYD3 and H3K4me3 occupancy of annotation genes. (A) SMYD3 expression level in tumor tissue following treatment with ZYZ384. (B) H3K4me3 expression level in tumor tissue after treatment with different dosages of ZYZ384. (C) Distribution of peaks at gene functional regions. (D) Relative reads of gene body distribution (E) KEGG enrichment analysis. (F) GO enrichment analysis. (G) Visualization of reads comparison. Statistical results of related genes data are expressed as mean ± SEM. GO, Gene Ontology; KEGG, Kyoto Encyclopedia of Genes and Genomes. *: *p* < 0.05; **: *p* < 0.01; one‐way ANOVA analysis.

In order to explore the antitumor mechanism of ZYZ384, the inhibitory effect of ZYZ384 on SMYD3 was determined in tumor tissues. The results indicated that SMYD3 expression was highly expressed in the control group but significantly diminished in the ZYZ384 treatment groups (Figure 5A). In parallel, the expression of H3K4me3 was also noted to decrease in a similar pattern to that of SMYD3 (Figure 5B). The tumor tissues from control and high‐dosage treatment group animals were isolated. Since SMYD3 can tri‐methylated H3K4, ChIP‐seq analysis was used. This method allowed for annotating H3K4me3 occupancy on genes (Figure 5C). Global H3K4me3 occupancy decreased near annotation genes' transcription start site (TSS; Figure 5D). H3K4me3 is often enriched at activated promoters near TSSs. When combined with Kyoto Encyclopedia of Genes and Genomes (KEGG) pathway enrichment analysis (Figure 5E) and Gene Ontology (GO) functional enrichment analysis (Figure 5F), attention focus on the AKT pathway, cell proliferation, and migration functions. Visualized reads and signal strength of H3K4me3 for the Rac1 gene at the promoter region decreased following treatment with ZYZ384 (Figure 5G). The H3K4me3 occupancy at the TSS of the Rac1 gene in tumor tissues was reduced by ZYZ384.

### 2.8 ZYZ384 impairs hepatocellular carcinoma tumor growth in vivo by reducing Rac1 and triggering cell cycle arrest via the AKT pathway

**FIGURE 6:**
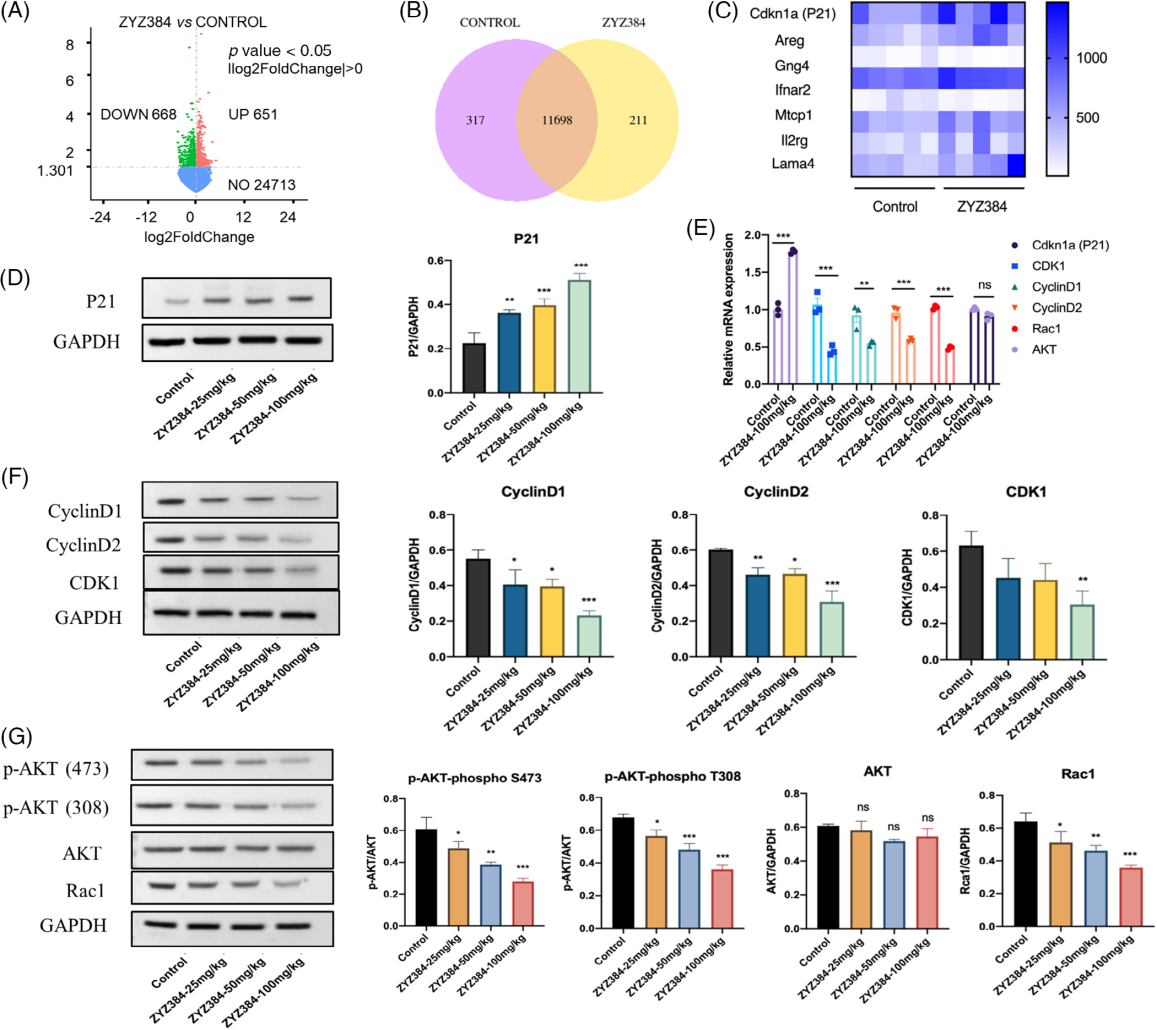
Molecular mechanism of ZYZ384 antitumor activity. (A) Differential gene volcano plot. (B) Venn diagram of gene coexpression. (C) Differentially gene expressed as presented as a clustering heat map. (D) P21 expression. (E) The mRNA level of related genes was assessed using quantitative real‐time reserve transcription polymerase chain reaction followed by (F, G) Western blot protein expression analysis. Statistical results of related genes data are expressed as mean ± SEM. *: *p* < 0.05; **: *p* < 0.01; ***: *p* < 0.001; one‐way ANOVA analysis.

To further investigate the mechanism, the RNA‐seq of the control and treatment group was also performed. RNA‐seq interrogation identified regulated genes (Figure 6A,B). The clustering heat map was used to show significant increases in Cdkn1a (P21; Figure 6C). This protein plays a key role in the cell cycle. The AKT pathway is likely involved in tumor proliferation and migration. The changes of cell cycle‐related genes AKT, P21, CDK1, CyclinD1, CyclinD2 in this signaling pathway were confirmed by q‐polymerase chain reaction (PCR) and Western blots analysis (Figure 6D–G). Both phosphorylation sites (473, 308) of AKT are changed (Figure 6G). Previous CHIP‐seq results revealed ZYZ384 reduced the H3K4me3 occupancy at the TSS of the AKT upstream Rac1 gene, the transcription and expression of the Rac1 gene are shown in Figure 6E,G. These results indicated that ZYZ384 impairs hepatocellular carcinoma tumor growth in vivo by reducing Rac1 and triggering cell cycle arrest via the AKT pathway.

## 3 DISCUSSION

The global rates of cancer incidence in the general population remain high, and there is a clear need to identify new therapeutic targets and drugs.^23^, ^24^ The histone methyltransferase, SMYD3, is overexpressed in many types of cancer(s) yet is often underexpressed in corresponding normal healthy tissues.^4^, ^5^ Moreover, SMYD3 is considered a biomarker for the poor prognosis of liver cancer,^25^, ^26^ and these traits make SMYD3 an ideal target for drug discovery initiatives. Therefore, the current research focuses on SMYD3 and uses a combination of bioinformatics, classical pharmacology, and screening to identify novel SMYD3 therapeutics.

Virtual screening and structural‐based drug design technologies, namely, various bioinformatics tools, including 3D structure information, has allowed researchers to study protein interactions with small molecular weight molecules.^17^, ^27^, ^28^, ^29^ Consequently, in the current work, computational docking, BLI analysis, and MD approaches were utilized in the current research. This integrated approach has allowed for the rapid analysis of small molecular compounds and the interrogation of interactions of candidate molecules with the SMYD3 protein. In addition, docking studies assisted in the rapid analysis of how selected compounds interacted with the SMYD3 protein at the amino acid residue level, allowing for the calculation of binding energies in protein–compound complexes.

Using these approaches, we developed and designed several novel compounds; one molecule, ZYZ384, has SMYD3 inhibition effect. Molecular docking and MD simulation showed that ZYZ384 binds strongly to SMYD3 at regions, SER‐182, PHE‐183, CYS‐186, MET‐190, ILE‐214, TYR‐257, LYS‐297, ASP‐332, HIS‐366, and VAL‐368, respectively. This interaction is mediated by extensive hydrogen bond formation between amino acid residues and ZYZ384, Van der Waals force interactions, and Pi–Pi conjugation. PHE‐183, CYS‐186, MET‐190, and VAL‐368 are critical residues for ZYZ384 action and activity. In vitro assessment indicated that ZYZ384 binds to the SMYD3 protein in a concentration‐dependent manner, reducing the activity of the SMYD3 enzyme. Moreover, when applied to various cancer cell lines, ZYZ384 significantly reduced the proliferation of cells. Among the cells tested, ZYZ384 showed a significant inhibitory effect on the proliferation of human hepatoma HepG2 cells, and these results informed the in vivo mouse model studies.

In mice, the maximum dose of ZYZ384 was administered by gavage (2 g/kg), and toxicology impacts were determined postadministration at 14 days. No animals died within 14 days, and no detrimental impacts of ZYZ384 treatment, namely, feeding or drinking responses, impacts on body weight, or behavioral changes, were reported. In the tumor‐bearing animals, ZYZ384 was found to inhibit the growth of implanted HepG2 tumors in nude mice correlating with the inhibition of SMYD3. RNA‐seq and ChIP‐seq were performed to explore the in‐depth antitumor mechanisms linked to ZYZ384. In epigenetic studies, H3K4me3 is enriched at promoters of activated genes near TSSs.^30^, ^31^, ^32^ ChIP‐seq revealed the H3K4me3 decreased at the promoter of the Rac1 gene following treatment with ZYZ384, which led to Rac1gene transcriptional repression. Rac1 is a member of the Rho GTPase family, which typically controls the assembly and disassembly of cytoskeletal elements.^33^ In addition, Rac1 activity is critical for many cellular activities, including phagocytosis, migration, adhesion, and differentiation of multiple cell types,^34^ and it also plays an important role in cell growth and cell cycle regulation.^35^ Importantly, the overactivation and overexpression of Rac1 are closely associated with aggressive tumor growth.^33^ Similarly, RNA‐seq revealed parallel changes in the AKT pathway. In many forms of human cancers, genes associated with the AKT pathway are most frequently altered. As a result, the aberrant activation of this pathway is associated with cellular transformation, tumorigenesis, cancer progression, and drug resistance.^36^ RNA‐seq indicated a significant increase of Cdkn1a (P21) after treatment of ZYZ384, Cell cycle‐related gene expressions have been evaluated and verified. GO functional annotation after treatment is also mainly enriched in the cell migration function, but the nude mice tumor‐bearing model cannot well simulate the invasion and migration phenotype of actual tumors, we will apply ZYZ384 on more alternative cancer models to explore its mechanism of action in the future.

We were granted a Chinese patent (CN114246851A) to protect the structure and activity of ZYZ384, the effectiveness of ZYZ384 at high concentrations presents a challenge for future clinical use. This is due to the poor water solubility of ZYZ384. Currently, we are trying to transform it into hydrochloride or culture cocrystallization to improve its solubility, bioavailability, and druggability. Drug resistance and combination therapy of ZYZ384 on other models will be gradually carried out in the follow‐up work. Although we developed a small molecule targeting SMYD3, we still need to figure out whether it is a selective inhibitor, and related research is to be continued.

Taken together, our study developed a novel molecule ZYZ384 targeting SMYD3 for hepatocellular carcinoma via reducing H3K4 trimethylation of the Rac1 promoter. ZYZ384 is a promising candidate drug that could be further developed to treat on cancer(s).

## 4 MATERIALS AND METHODS

### 4.1 Materials

Reagents were obtained from the following: lead compounds were purchased from Guangzhou BaoYan Technology Co., Ltd. Company. A series of novel small molecules were synthesized by our group at the State Key Laboratory (SKL) Macau and verified by NMR. Dulbecco's modified eagle medium (DMEM), penicillin/streptomycin, 0.25% Trypsin‐EDTA, and fetal bovine serum (FBS) were all purchased from Gibco. The SMYD3 Homogeneous Assay Kit and purified SMYD3 N‐terminal GST tag protein was purchased from BPS Bioscience. MTT (3‐(4,5‐Dimethylthiazol‐2‐yl)‐2,5‐diphenyltetrazoliumromide) powder was obtained from Thermo Fisher. Sorafenib was got from MedChemExpres. SMYD3, H3, H3K4me3, P21, CyclinD1, CyclinD2, CDK1, Rac1, AKT, p‐AKT (308, 473), and Glyceraldehyde‐3‐phosphate dehydrogenase (GAPDH) primary antibodies were all purchased from Abcam or Santa Cruz.

### 4.2 Structure‐based virtual screening

The structure of SMYD3 was derived from the Protein DataBank (PDB ID: 5CCL) and prepared with the Protein Preparation Wizard in Maestro (Schrodinger 2015) including removal of water and ions, protonation, addition of missing atoms, completion of missing groups, and use of OPLS3e force field for constraint minimization.^37^ For the ligands, a total of 211,838 compounds from Specs databases were prepared using the property calculator module in the Schrödinger Maestro software, including calculating the physical and chemical properties of the small molecule compound library data and filtering by the Lipinski rules, protonation, and energy minimization. Before the virtual screening, it was necessary to dock the original ligand in the complex to investigate the docking method's reliability and then select the protein's original ligand as the centroid of the 10 Å box. Standard precision (SP) and extra precision (XP) methods were used to screen compound databases and construct the compound ranking score.

### 4.3 Structure modification based on lead compound 1

In the structural design, based on the indolin‐2‐one structure of the lead compound 1, the Schiff base was added by Mannich reaction to obtain compounds. The Schiff hydrazone (C = N–) framework is an exceptionally adaptable drug‐like moiety that has recently been utilized in the development of cancer(s) treatments or cellular apoptosis.^38^ The structures containing this bioactive moiety showed remarkable anticancer activities via the restraint of numerous kinds of enzymes, proteins, and/or receptors that play essential roles in cell growth and survival.^39^ Consequently, Schiff‐fused chemical compounds, because of their wide scope of biological activities and synthetic applications, have been developed as a target heterocyclic framework in the research and development of medicinal chemistry. The SM series synthesis method (general procedure) is as follows:

A mixture of isatin (2, 0.1 mM) and hydrazine hydrate (1.2 Equiv.) in methanol was refluxed for 3 h and cooled to room temperature (r.t.). The precipitate was filtered, washed with cold methanol, and dried at r.t. in the open air to give the hydrazine‐derivative compound 3 with a 96% yield. A mixture of compound 3 (1.61 g, 10 mM) and the appropriate aromatic aldehyde (10 mM) in methanol (80 mL) and acetic acid (0.5 mL) was refluxed for 10 h. After standing at r.t. for 1 h, the product obtained was filtered and crystallized from methanol to generate 4a–e.

1H‐indene‐1,3(2H)‐dione was used as raw material in the design and synthesis of Schiff base analogs via the Mannich reaction. These compounds are named the SMI series. The general procedure for synthesis is as follows:

A mixture of 1,3‐Indanedione (5, 2 mM), methyl iodide (6 mM), and potassium fluoride (10 mM) in anhydrous acetonitrile (2 mL) was stirred at 70°C under nitrogen gas for 24 h. After the reaction was completed, the solution was cooled to r.t., and the mixture was extracted with dichloromethane. The organic layers were then pooled and dried over anhydrous Na_2_SO_4_ before being concentrated under reduced pressure. The residue was purified by silica gel column chromatography to give the 2,2‐dimethylindan‐1,3‐dione (6, 84%). Subsequently, compound 6 (174.1 mg, 1 mM) was added to a solution of appropriate amines (2.5 mM) and potassium fluoride (0.24 mL,10 mM) in anhydrous toluene (2 mL). Two drops of *p*‐toluenesulfonic acid were added to the solution with constant stirring. Then the reaction mixture allowed to proceed under reflux conditions for 8 h. After the reaction was completed, the mixture was cooled to r.t. via the addition of water and extraction with dichloromethane. The combined organics layers were dried over anhydrous Na_2_SO_4_ and concentrated in vacuo. The residue was purified using silica column chromatography to obtain molecules.

### 4.4 Biolayer interferometry (BLI) assay

The SMYD3‐GST tag protein (BPS Bioscience) was immobilized onto a GST biosensor (Fortebio). ZYZ384 was tested across the concentrations range of 6.25–200 µM. After baseline setup using Phosphate buffer saline (PBS), the biosensor tips were immersed in wells containing ZYZ384 continuous diluent for 180 s to combine, followed by a 180‐s dissociation step. They calculated *K*_D_ value with a 1:1 model in data analysis software 9.0 (Fortebio).

### 4.5 Molecular docking studies

To determine molecule selectivity, the optimal binding mode and interaction of ZYZ384 with SMYD3 protein were assessed by molecular docking techniques using the CDOCKER module of Discovery Studio version 4.5 (Dassault Systemes BIOVIA) software.^40^ The structure of ZYZ384 was produced using Chemdraw software, and docking runs was performed and analyzed. A ranked list of the docked molecule to protein target was assessed. The ZYZ384 protein complex that best represented the docked complex was utilized for further analysis using the PyMol (Version 2.5.2) molecular visualization platform.^41^

### 4.6 Molecular dynamic simulation study

The stability of the ZYZ384 interaction and binding to the SMYD3 active site was determined by MD simulation with the 2019.3 package GROMACS version.^42^ The amber99sb‐ildn force field was applied to protein parameters. Multiwfn was used to calculate ZYZ384 Electrostatic Potential^43^ and parameterized with the General Amber Force Field (GAFF)^44^ to allocate atom types and bonding parameters. Two sodium ions were added to neutralize the charge of the system, and the long‐range electrostatic interactions were calculated using the Particle Mesh Ewald (PME) method.^45^ Proteins were solvated in a 12‐layer cubic box with periodic boundary conditions using the TIP3P water model with a minimum distance of 1.0 Å from solute to box boundary. Prior to simulation, the two systems were first balanced through 0.1 ns in the normal volume and temperature (NVT), with normal pressure and temperature (NPT) gathering at 300 K and 1 atmosphere, using periodic boundary conditions to apply position constraints on proteins and small molecules. Subsequently, a 100 ns MD production run was performed in the NPT assembly using a Berendsen thermostat–barostat. The periodic boundary condition is used in the system, the motion equation is used together with the leap‐frog algorithm, and the time step is 2 fs. Various geometric properties of the system, such as RMSD and RMSF, were determined and graphed using the Xmgrace tool. The gmmpbsa method was applied to calculate binding free energy and energy decomposition.^46^

### 4.7 SMYD3 homogeneous assay

The commercial SMYD3 homogenous assay kit is designed to detect SMYD3 activity for screening and analytical applications. The SMYD3 homogenous assay kit is available in a convenient AlphaELISA format with GST‐labeled SMYD3 substrate, primary antibody, methylation detection buffer, and purification of SMYD3 for 384 enzyme reactions. The key to the SMYD3 homogenous assay is a particular antibody that recognizes methylated substrates. Using the kit, methyltransferase can be detected in three simple steps on a microtitration plate. First, samples containing the SMYD3 enzyme and ZYZ384 in different dosages were incubated in substrates for 3 h. Next, the receptor bead and primary antibody are added, the donor bead is added, and the Alpha counts are recorded.

### 4.8 Cell culture

Human HepG2, A549, HCT116, MDA‐MB‐231, and Miapaca2 cells were purchased from American Type Culture Collection (ATCC) and cultured in DMEM medium supplemented with 1% penicillin/streptomycin and 10% FBS at 37°C under 5% CO_2_ atmosphere and 95% air. Trypsin EDTA was used for cell passage.

### 4.9 Cytotoxicity analysis

Cell viability was assessed in several cancer cell lines and determined using the MTT assay in a 96‐well plate format. In brief, cells from 100 mL medium (1 × 10^4^/well) were inoculated in 96‐well plates and treated with ZYZ384. After 24 h of treatment, cell viability was determined by adding 1 mg/mL MTT‐containing medium for 4 h, followed by 100 mL Dimethyl sulfoxide (DMSO) to dissolve formazan. The absorbance at 570 nm was recorded using a microplate ultraviolet/visible (UV/VIS) spectrophotometer (Tecan).

### 4.10 Cell transfection

SMYD3 overexpression (SMYD3 OE) plasmid (Sino Biological) was mixed with lipofectamine 2000 for plasmid transfection. After transfection for 6 h, cells were exposed to the indicated ZYZ384 treatments for another 24 h.

### 4.11 Animal experiment

#### 4.11.1 Animal care

Six‐week‐old male nude mice and C57BL/6 mice were obtained from Chongqing Tengxin Biotechnology Co. LTD and free access to food and water in specific pathogen‐free (SPF) rooms with 25°C, 50% relative humidity, and 12‐h light /12‐h dark cycle.

#### 4.11.2 Short‐term toxicity study of C57BL/6 mice

C57BL/6 mice were randomly divided into two groups (*n* = 10) to determine the short‐term toxicity of ZYZ384. Mice were given the maximum dose of 2 g/kg orally administered ZYZ384 once, while the control group was treated with the same volume of sterilized water. The health conditions of all mice were monitored for 2 weeks.

#### 4.11.3 The establishment and treatment of the tumor‐bearing HepG2 nude mice

The tumor‐bearing nude mice model was established by direct injection of hepG2 cells into the left armpit of nude mice. Animals were subsequently divided into five groups (*n* = 7). All compounds were applied via intragastric administration, and sorafenib was used as the positive control. Other groups, except the control group, were treated with different concentrations of ZYZ384. Body weight and tumor volume were monitored and recorded throughout the treatment period.

### 4.12 RNA‐seq and ChIP‐seq analysis

#### 4.12.1 RNA‐seq

Sample preparation and sequencing: Control and ZYZ384 (100 mg/kg) groups of tumor tissue (*n* = 5) RNA integrities were assessed using the RNA Nano 6000 Bioanalyzer 2100 system (Agilent Technologies), Library preparation for transcriptome sequencing, Clustering, and sequencing on a cBot Cluster Generation System using TruSeq PE Cluster Kit v3‐cBot‐HS (Illumina).

Data analysis: Quality control, the reference genome reads mapping, novel transcripts prediction, quantification of gene expression level, differential expression analysis, GO and KEGG enrichment analysis.

#### 4.12.2 ChIP‐seq (H3K4me3)

Sample preparation and sequencing: Control and ZYZ384 (100 mg/kg) groups of tumor tissue (*n* = 3). ChIP DNA degradation and contamination were monitored on agarose gels. DNA purity was checked using the NanoPhotometer® and spectrophotometer (IMPLEN). DNA concentration was measured using Qubit® DNA Assay Kit in Qubit® 3.0 Fluorometer (Life Technologies), and the purified DNA was used for ChIP‐seq library preparation. The library was constructed by Novogene Corporation. Subsequently, pair‐end sequencing of the sample was performed on the Illumina platform (Illumina).

Data analysis: Quality control reads mapping to the reference genome, fragment size estimation, peak detection, motif analysis, peak annotation, and different peak analyses.

### 4.13 Quantitative real‐time polymerase chain reaction (qRT‐PCR) analysis

Total tumor tissue mRNA was extracted using TRIzol reagent (Invitrogen), and NanoDrop™ spectrophotometer (Thermo Scientific) quantification. A Biotool cDNA Synthesis Kit (Biotool) produced cDNA. Target gene expression was measured by semiquantitative q‐PCR using iTaqTM Universal SYBR Green Supermix (Bio‐Rad) on ViiA™ 7 Real‐Time PCR System (Applied Biosystems). β‐Actin served as the reference gene. The 2 − △△Ct method was used to analyze the relative target gene expression. The gene‐specific primer sequences used in this work are listed in Table S2.

### 4.14 Western blot analysis

Isolated protein (30 µg) was obtained from tissues, separated on a sodium dodecyl sulfate‐polyacrylamide gel electrophoresis (SDS‐PAGE) gel, and transferred to a polyvinylidene fluoride membrane. Membranes were then incubated in 5% Bovine Serum Albumin (BSA). The membranes were then probed with antibodies against SMYD3, H3, H3K4me3, P21, CyclinD1, CyclinD2, CDK1, Rac1, AKT, p‐AKT (308, 473), and GAPDH and incubated with horseradish peroxidase‐conjugated goat antirabbit or antimouse antibodies. The immunoreactive protein is visualized by enhanced chemiluminescence, and the signal intensity was detected and quantified using ImageJ.

The antibodies Anti‐p21(ab109520), Anti‐Cyclin D1 (ab134175), Anti‐Cyclin D2(ab207604), Anti‐CDK1(ab131450), H3 (ab267372), H3k4me3 (ab213224), Anti‐pan‐AKT (phospho T308)(ab8933), Anti‐AKT (phospho S473) (ab285140), pan‐AKT (ab18785), Anti‐Rac1(ab155938), and Anti‐SMYD3 (ab228015) are purchased from Abcam; SMYD3(C3): sc‐398085 and GAPDH sc‐32233 are purchased from Santa Cruz.

### 4.15 Statistical analysis

All experimental results except high‐throughput sequence data were expressed as mean ± SEM. All data were analyzed using GraphPad Prism software 8.0. One‐way ANOVA determined statistical significance between multiple groups. *p* value <0.05 was considered statistically significant.

## AUTHOR CONTRIBUTIONS

**Qian Ding**: Designed experiments. **Qian Ding; Jianghong Cai; Li Jin; and Wu**: Song performed experiments. **Wei Hu and Wei Lei**: Gave valuable suggestions on the research. **Qian Ding**: Analyzed data. **Qian Ding and Jianghong Cai**: Wrote the manuscript. **Peter Rose**: Checked the manuscript. **Zhiyuan Tang; Yangyang Zhan; and Leilei Bao**: Provided valuable opinions when revising the article and helped process the figures. **Yi Zhun Zhu**: Supervised this work. All the authors have read and approved the final manuscript.

## CONFLICT OF INTEREST STATEMENT

The authors declare no conflicts of interest.

## ETHICS STATEMENT

Special Approval number: MUST‐SA‐20240221001.

Approved by Medical Ethics Committee Macau University of Science and Technology.

Statements:

In the work entitled “A novel small molecule ZYZ384 targeting SMYD3 for hepatocellular carcinoma via reducing H3K4 trimethylation of the Rac1 promoter.” These carcinoma xenograft mice were originally planned to be treated with ZYZ384 for 30 days. During the experiment, we observed and supposed that the tumor volume may be too large on the 30th day, so we terminated the experiment on the 24th day of treatment in advance. After removing and measuring the tumors, we found some exceeded the limit. However, considering that ZYZ384 is a brand‐new structure that can reduce the growth of solid tumors, it is of great significance for future cancer treatment, to avoid repeated animal experiments causing more animal suffering, we apply special ethical approval for this experiment.

## Supporting information

Supporting Information

## Data Availability

The data are available from the corresponding author on reasonable request.
