# Supplementary material for: A novel small molecule ZYZ384 targeting SMYD3 for hepatocellular carcinoma via reducing H3K4 trimethylation of the Rac1 promoter
Source: MedComm (2020). 2024 Sep 15;5(10):e711. doi: 10.1002/mco2.711 (PMC11401973; doi:10.1002/mco2.711)
Supplement: Supplementary file 1 — Supporting Information [file MCO2-5-e711-s001.docx]

**supplementary DATA**

**A Novel Small Molecule ZYZ384 Targeting SMYD3 for Hepatocellular Carcinoma via Reducing H3K4 Trimethylation of the Rac1 Promoter**

Qian Ding^1,2,3#^，Jianghong Cai ^1#^ , Li Jin^1^ , Wei Hu^1^, Wu Song^1^, Peter Rose^4^ , [Zhiyuan Tang](https://pubmed.ncbi.nlm.nih.gov/?sort=date&term=Tang+Z&cauthor_id=32143183)^6^, Yangyang Zhan^7^, Leilei Bao^7^, Wei Lei^2^ and Yi Zhun Zhu ^1, 5*^

^1^ State Key Laboratory of Quality Research in Chinese Medicine & (R & D Center) Lab. for Drug Discovery from Natural Resource & School of Pharmacy, Macau University of Science and Technology, Macau SAR 999078, China.

^2^ Affiliated Hospital of Guangdong Medical University, Zhanjiang, 524000, China.

^3^ School of Basic Medicine, Guizhou University of Traditional Chinese Medicine, Guiyang 550000, China.

^4^ School of Biosciences, University of Nottingham, Loughborough, United Kingdom.

^5^ Shanghai Key Laboratory of Bioactive Small Molecules, Department of Pharmacology, School of Pharmacy, Fudan University, Shanghai 201203, China.

^6^ Department of Pharmacy, Affiliated Hospital of Nantong University & Medical School of Nantong University, Nantong 226001, China.

^7^ Department of Pharmacy, Shanghai Eastern Hepatobiliary Surgery Hospital, Navy Military Medical University, 225 Changhai Road, Yangpu District, Shanghai, China.

^#^ These authors contributed equally to this work.

* Correspondence:

Chair Prof. Yi Zhun Zhu

E-mail: [yzzhu@must.edu.mo](mailto:yzzhu@must.edu.mo)

**1. Virtual Screening verified lead compound**.

Prior to conducting a full screening, the binding mode for the small molecule ligand to the active site of the SMYD3 protein was determined ( Figure S1. A and B); the key residues THR-184, GLU-192, SER-202, TYR-239 play an essential role in stabilizing the ligand-protein complex (Figure S1. B and C). The known binding position of the original ligand was consistent with the previous complex data (Figure S1.D) and showed that our virtual screening methods are effective. Using the virtual system, we determined the binding position of each target molecule to SMYD3 and allowed for the characterization of the top-ranked lead compound 1. This molecule was selected for further experimental validation (Table S1).


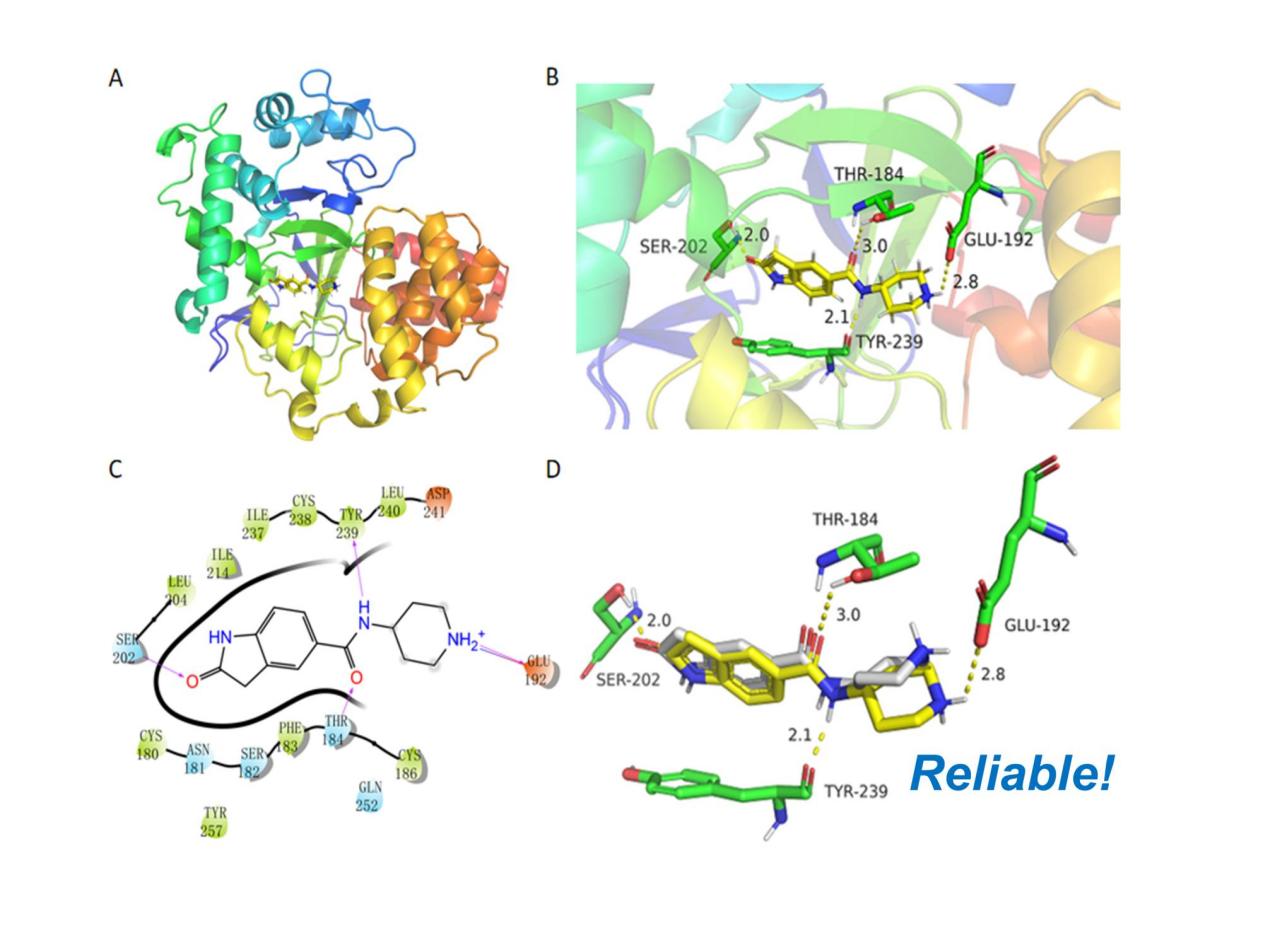


**Figure S1. Analysis of the effectiveness of virtual screening methods.**

1. The overall 3D structure of the SMYD3 ligand complex. (B) A close view of the active site binding with a natural ligand. (C) The 2D protein-ligand interaction diagram of the ligand - SMYD3 complex. (D) The re-docking results of active compounds with the target.

**Table S1. Molecular Docking Score of Top 10 Compounds**

| Name | CAS | Molecular_weight | Binding energy /kcal/mol | Hydrogen bond acceptor | Hydrogen bond donor | The rotating bond |
| --- | --- | --- | --- | --- | --- | --- |
| NS1619 | 153587-01-0 | 362.231 | -11.2133 | 2 | 2 | 3 |
| Dovitinib (TKI258) | 915769-50-5 | 392.43951 | -10.6645 | 4 | 4 | 4 |
| LIT927 | 2172879-52-4 | 328.755 | -10.4193 | 4 | 2 | 3 |
| ASP9521 | 1126084-37-4 | 330.32001 | -10.053 | 3 | 2 | 5 |
| Diosmetin | 520-34-3 | 300.27045 | -9.8761 | 5 | 3 | 2 |
| Piceatannol | 10083-24-6 | 244.24934 | -9.79581 | 4 | 4 | 2 |
| Clevudine (Levovir) | 163252-36-6 | 260.22391 | -9.6853 | 5 | 3 | 3 |
| Tenatoprazole | 113712-98-4 | 346.41086 | -9.67032 | 6 | 3 | 5 |
| CH5183284 | 1265229-25-1 | 356.38901 | -9.94544 | 4 | 4 | 3 |
| **Original ligand** | 1519233-58-9 | 259.308 | -7.9623 | 2 | 2 | 3 |

**2. Spectra data for intermediates and compounds 4a-e, 7a-c, and 8a-b:**

Structure modification is based on the lead compound (NS1619) with the lowest binding energy from virtual screening results, and then refers to structures of the existing SMYD3 inhibitor, introducing the reported “anti-caner” framework (schiff hydrazone (C=N-) ), after the reasonable and feasible design of the synthetic route and computer-aided ADMET prediction calculation, finally design and successfully synthesize two series of novel SMYD3 small molecules. The HRMS, 1H NMR, and 13C NMR spectra for intermediates and compounds of the two series of novel small molecules are shown as follows.

(Z)-3-hydrazonoindolin-2-one（**3**）(Intermediate of SM series)

^1^H NMR (600 MHz, DMSO) δ 10.76 (s, 1H), 10.58 (d, *J* = 14.7 Hz, 1H), 9.56 (d, *J* = 14.5 Hz, 1H), 7.43 (d, *J* = 6.3 Hz, 1H), 7.22 (dd, *J* = 7.7, 1.2 Hz, 1H), 7.06 -7.04 (m, 1H), 6.96 (s, 1H). ^13^C NMR (151 MHz, DMSO) δ 163.20, 127.58, 126.69, 122.46, 117.91.

2,2-dimethyl-1H-indene-1,3(2H)-dione (**6**) (Intermediate of SMI series)

^1^H NMR (600 MHz, DMSO) δ 8.05-7.97 (m, 4H), 1.21 (s, 6H). ^13^C NMR (151 MHz, DMSO) δ 203.80, 139.78, 136.48, 123.40, 49.18, 20.03.

(E)-3-(((E)-2-(trifluoromethoxy)benzylidene)hydrazono)indolin-2-one (**4a**)

^1^H NMR (600 MHz, CDCl_3_) δ 8.95 (s, 1H), 8.33 (d, *J* = 7.8 Hz, 1H), 8.15 (s, 1H), 8.06 (d, *J* = 7.6 Hz, 1H), 7.59 (t, *J* = 7.9 Hz, 1H), 7.48 (t, *J* = 7.6 Hz, 1H), 7.41- 7.37 (m, 2H), 7.07 (d, *J* = 7.6 Hz, 1H), 6.91 (d, *J* = 7.6 Hz, 1H). ^13^C NMR (151 MHz, CDCl_3_) δ 165.09, 156.20, 150.93, 143.47, 133.75, 133.25, 129.84, 128.34, 127.36, 126.70, 123.33, 121.51, 117.20, 110.70, 29.71, 14.13.

(E)-3-(((E)-2-ethylbutylidene)hydrazono)indolin-2-one (**4b**)

^1^H NMR (600 MHz, CDCl_3_) δ 11.17 (d, *J* = 6.8 Hz, 1H), 8.51- 8.44 (m, 1H), 7.47 (d, *J* = 7.6 Hz, 1H), 7.08 (d, *J* = 7.7, 1.4 Hz, 1H), 6.97 – 6.94 (m, 1H), 6.79 (d, *J* = 7.7 Hz, 1H), 1.75 – 1.46 (m, 5H), 0.93 (s, 6H). ^13^C NMR (151 MHz, CDCl_3_) δ 169.56, 163.89, 148.67, 141.43, 131.56, 127.93, 121.45, 115.54, 108.81, 44.59, 27.61, 23.02, 9.93.

1. -3-(((E)-(3-fluoropyridin-4-yl) methylene) hydrazono)indolin-2-one (**4c**)

^1^H NMR (600 MHz, CDCl_3_) δ 9.56 (d, *J* = 2.3 Hz, 1H), 8.77 (s, 1H), 8.44 (s, 1H), 8.13 (d, *J* = 8.6 Hz, 2H), 8.03 (s, 1H), 7.90 (d, *J* = 8.1 Hz, 1H), 7.79 -7.76 (m, 1H), 7.60-7.58 (m, 1H), 7.33 (dd, *J* = 7.8, 1.3 Hz, 1H), 7.02 (dd, *J* = 7.6, 0.9 Hz, 1H), 6.83 (s, 1H). ^13^C NMR (151 MHz, CDCl_3_) δ 164.10, 159.44, 150.41, 148.08, 142.44, 137.19, 132.85, 130.55, 129.03, 128.67, 126.72, 126.48, 125.63, 122.45, 116.27, 109.68.

(E)-3-(((E)-quinolin-3-ylmethylene)hydrazono)indolin-2-one **(4d**)

^1^H NMR (600 MHz, CDCl_3_) δ 8.68 (s, 1H), 8.59 (d, *J* = 1.8 Hz, 1H), 8.55 (d, *J* = 5.0 Hz, 1H), 7.96- 7.92 (m, 2H), 7.82 (d, *J* = 7.6 Hz, 1H), 7.35 (d, *J* = 7.8, 1.3 Hz, 1H), 7.01 (dd, *J* = 7.7, 1.0 Hz, 1H), 6.85 (d, *J* = 7.9 Hz, 1H). ^13^C NMR (151 MHz, CDCl_3_) δ 163.86, 157.72, 155.96, 150.30, 149.46, 145.19, 142.79, 138.76, 133.29, 128.65, 122.46, 119.59, 115.78, 109.98.

(E)-3-(((E)-thiophen-2-ylmethylene)hydrazono)indolin-2-one (**4e**)

^1^H NMR (600 MHz, CDCl_3_) δ 8.90 (s, 1H), 8.29 (d, *J* = 7.6 Hz, 1H), 7.82 – 7.76 (m, 1H), 7.64 (d, *J* = 5.0 Hz, 1H), 7.58 (d, *J* = 3.7 Hz, 1H), 7.37 (t, *J* = 7.7 Hz, 1H), 7.21- 7.18 (m, 1H), 7.10 (t, *J* = 7.7 Hz, 1H), 6.88 (d, *J* = 7.8 Hz, 1H). ^13^C NMR (151 MHz, CDCl_3_) δ 165.64, 158.68, 151.63, 145.74, 143.22, 139.06, 134.43, 133.38, 132.40, 130.29, 128.39, 123.40, 117.61, 110.50.

(Z)-3-((4-methoxyphenyl)imino)-2,2-dimethyl-2,3-dihydro-1H-inden-1-one (**7a**)

^1^H NMR (600 MHz, CDCl_3_) δ 7.99 (d, *J* = 2.5 Hz, 1H), 7.92 (d, *J* = 7.6 Hz, 1H), 7.86 (d, *J* = 2.6 Hz, 1H), 7.61 – 7.36 (m, 2H), 6.95 (s, 1H), 6.80 (d, *J* = 4.5 Hz, 2H), 3.87 (s, 3H), 1.31 (s, 6H). ^13^C NMR (151 MHz, CDCl_3_) δ 204.54, 156.51, 152.84, 140.38, 135.87, 134.76, 132.47, 127.04, 124.17, 123.65, 119.01, 116.45, 114.96, 114.83, 55.76, 22.69.

(Z)-2,2-dimethyl-3-(m-tolylimino)-2,3-dihydro-1H-inden-1-one (**7b**)

^1^H NMR (600 MHz, CDCl_3_) δ 7.92 (d, *J* = 7.6 Hz, 1H), 7.56 (t, *J* = 7.4 Hz, 1H), 7.39 (t, *J* = 8.3 Hz, 1H), 7.28 (t, *J* = 7.6 Hz, 2H), 7.00 (d, *J* = 7.7 Hz, 1H), 6.73 (d, *J* = 7.9 Hz, 1H), 6.69 (d, *J* = 14.7 Hz, 2H), 2.37 (s, 3H), 1.59 (s, 6H). ^13^C NMR (151 MHz, CDCl_3_) δ 206.41, 139.63, 138.87, 138.78, 134.78, 132.48, 129.52, 127.20, 124.66, 124.15, 118.33, 114.73, 49.41, 22.67.

(Z)-3-((4-(tert-butyl)phenyl)imino)-2,2-dimethyl-2,3-dihydro-1H-inden-1-one (**7c**)

^1^H NMR (600 MHz, CDCl_3_) δ 7.92 (d, *J* = 7.5 Hz, 1H), 7.56 (t, *J* = 7.5 Hz, 1H), 7.40 (d, *J* = 8.3 Hz, 2H), 7.38-7.32 (m, 1H), 6.79 (d, *J* = 8.2 Hz, 2H), 6.72 (d, *J* = 8.0 Hz, 1H), 1.43 (s, 6H), 1.37 (s, 9H). ^13^C NMR (151 MHz, CDCl_3_) δ 206.52, 170.50, 148.89, 147.01, 138.74, 134.75, 132.38, 127.06, 126.43, 125.32, 124.11, 118.27, 117.34, 49.47, 31.45, 22.68.

(1Z,3Z)-N1,N3-bis(4-methoxyphenyl)-2,2-dimethyl-1H-indene-1,3(2H)-diimine (**8a/ZYZ-384**)

^1^H NMR (600 MHz, CDCl_3_) δ 8.12 (d, *J* = 8.4 Hz, 2H), 7.88 (s, 2H), 7.49 (s, 2H), 7.32-7.28 (m, 2H), 7.01 (d, *J* = 7.7, 1.0 Hz, 2H), 6.83 (d, *J* = 7.8 Hz, 2H), 1.98 (s, 3H), 1.19 (t, *J* = 7.1 Hz, 9H). ^13^C NMR (151 MHz, CDCl_3_) δ 164.75, 154.98, 150.13, 146.72, 143.85, 142.27, 132.40, 128.87, 122.22, 122.07, 116.47, 109.63, 106.86, 59.40, 28.69, 20.05.

(1Z)-2,2-dimethyl-N1,N3-di-m-tolyl-1H-indene-1,3(2H)-diimine (**8b**)

^1^H NMR (600 MHz, CDCl_3_) δ 7.21- 7.15 (m, 4H), 7.03 (dd, *J* = 6.0, 3.2 Hz, 2H), 6.89 (d, *J* = 7.6 Hz, 2H), 6.66 (dd, *J* = 6.0, 3.2 Hz, 2H), 6.60 (d, *J* = 6.9 Hz, 2H), 2.28 (s, 6H), 1.48 (s, 6H). ^13^C NMR (151 MHz, CDCl_3_) δ 171.04, 150.90, 136.10, 130.27, 128.39, 126.44, 123.11, 117.44, 113.84, 47.58, 24.24, 20.47.

1. **HRMS, ^1^H NMR, and ^13C^ NMR spectra for intermediates and compounds 4a-h, 7a-c, and 8a-b:**


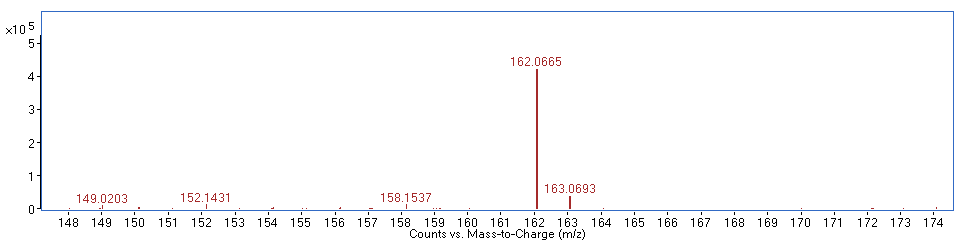


**Figure S2.** HRMS spectrum of Intermediate of SM series **(** **3)**


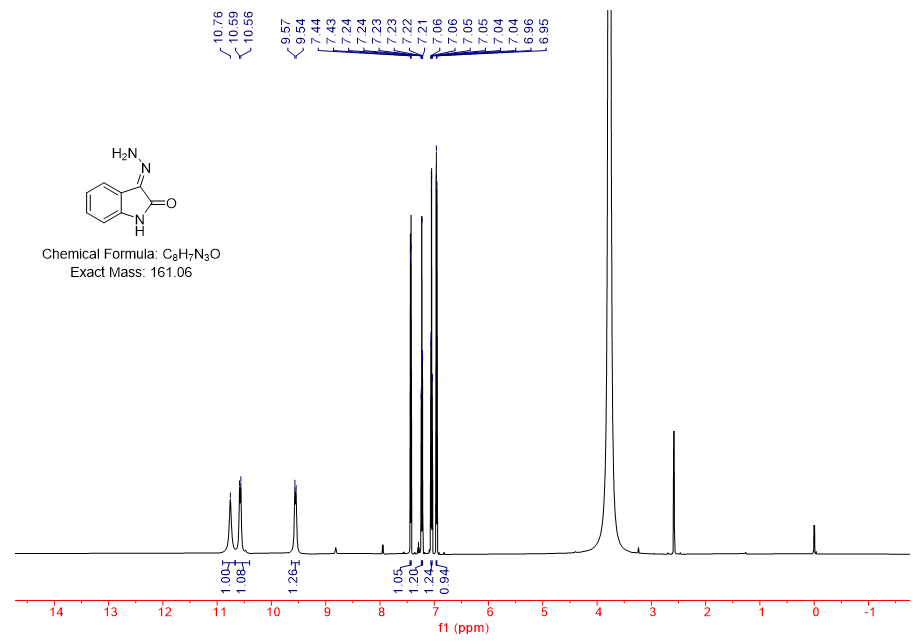


**Figure S3.** ^1^H NMR spectrum of Intermediate of SM series**(** **3)**


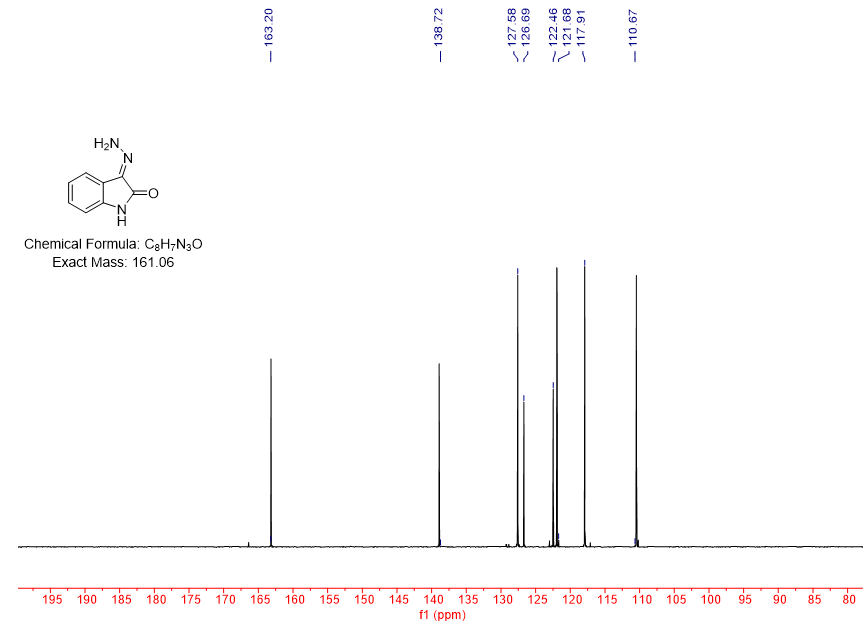


**Figure S4.** ^13^C NMR spectrum of Intermediate of SM series**( 3)**


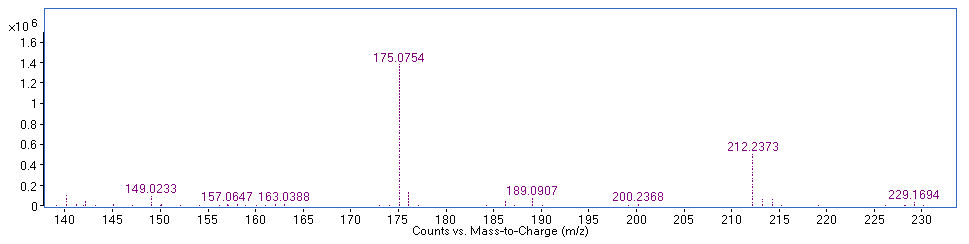


**Figure S5.** HRMS spectrum of Intermediate of SMI series **(6)**


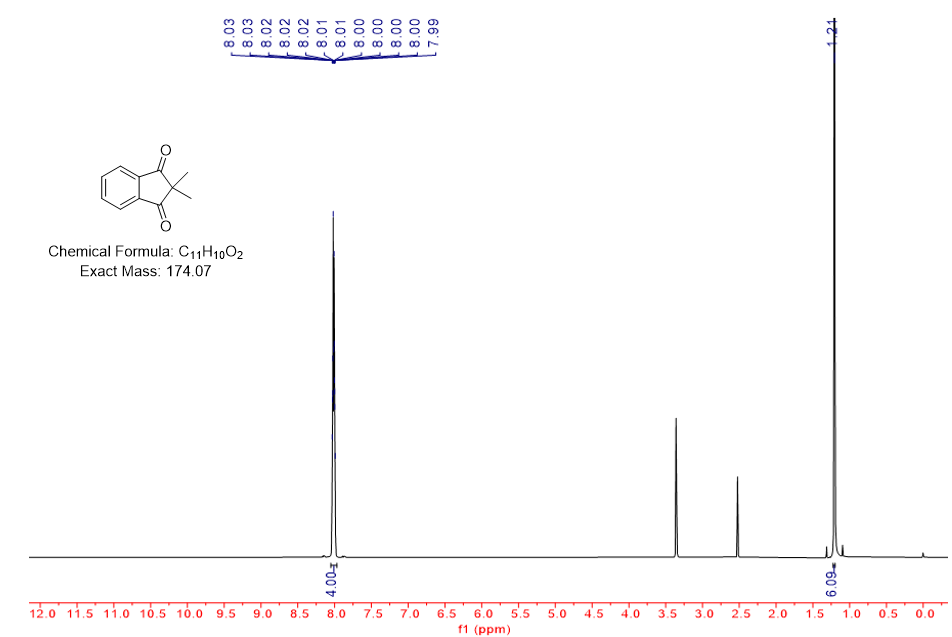


**Figure S6.** ^1^H NMR spectrum of Intermediate of SMI series **(6)**


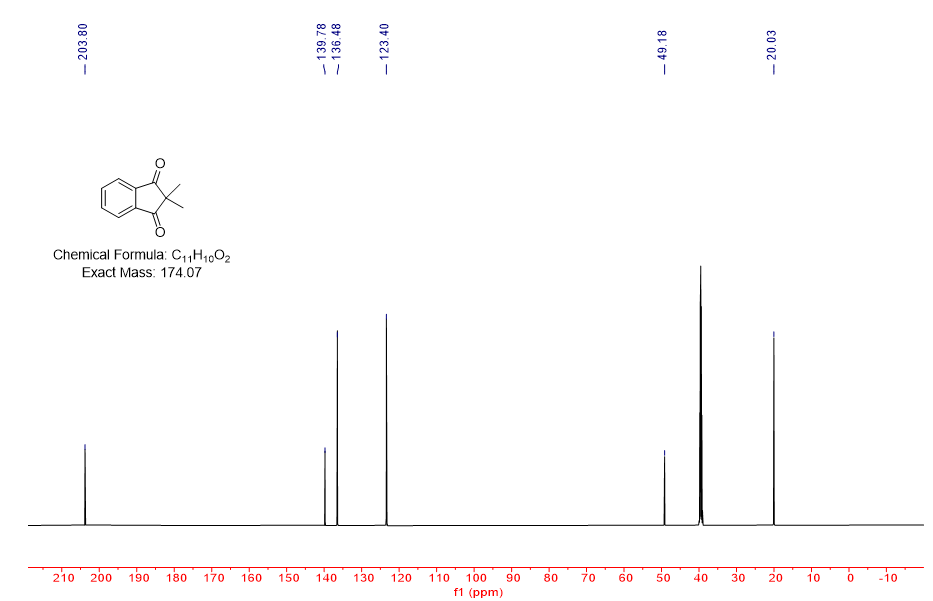


**Figure S7.** ^13^C NMR spectrum of Intermediate of SMI series **(6)**


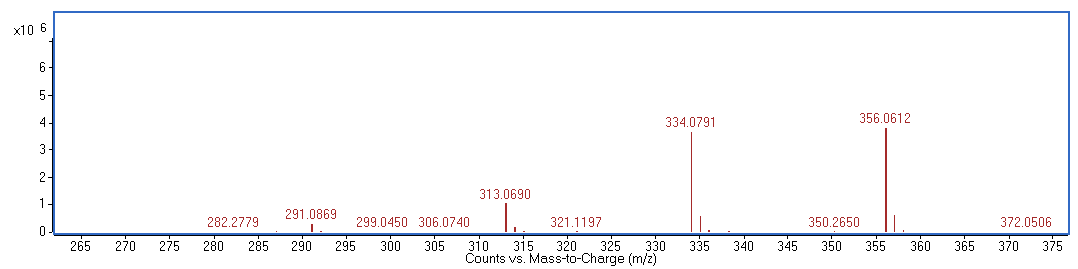


**Figure S8.** HRMS spectrum of **4a**


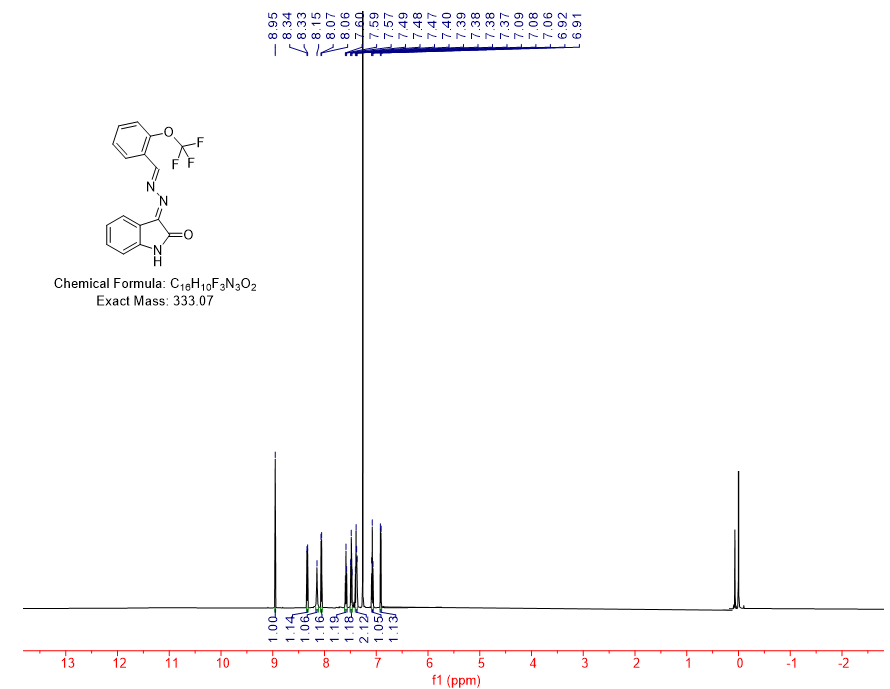


**Figure S9.** ^1^H NMR spectrum of **4a**


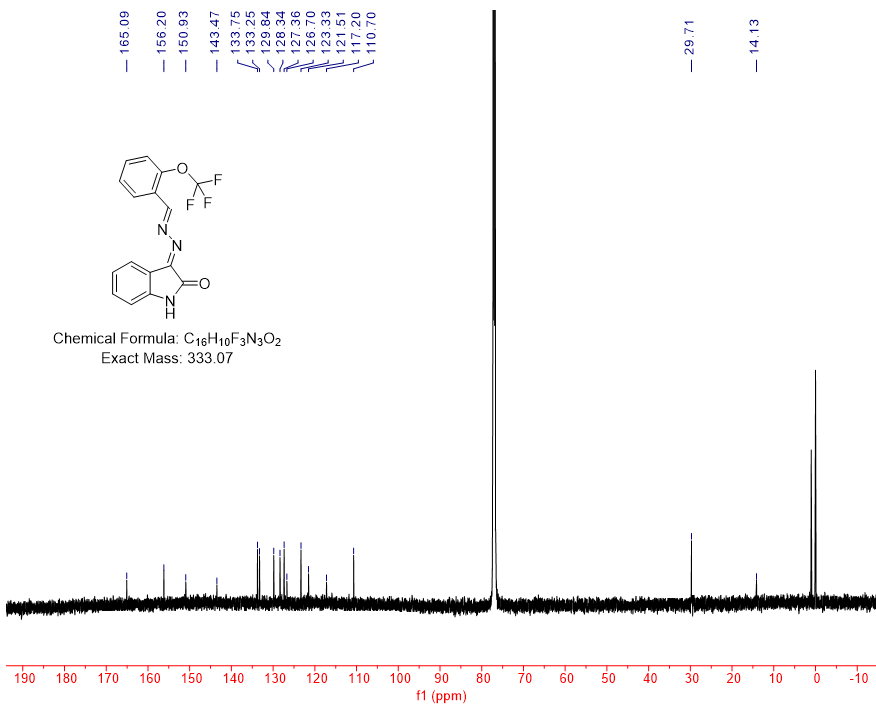


**Figure S10.** ^13^C NMR spectrum of **4a**


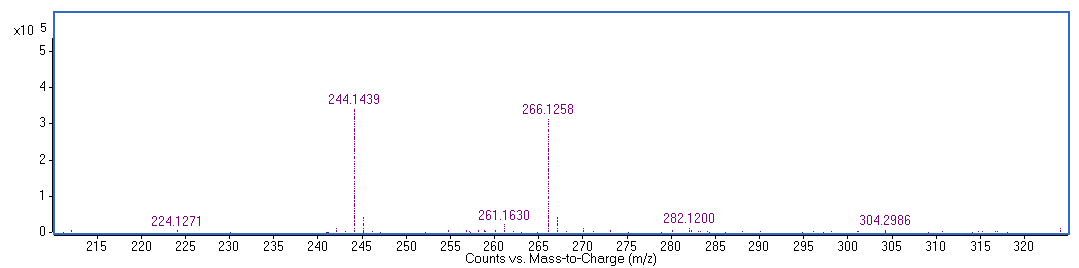


**Figure S11.** HRMS spectrum of **4b**


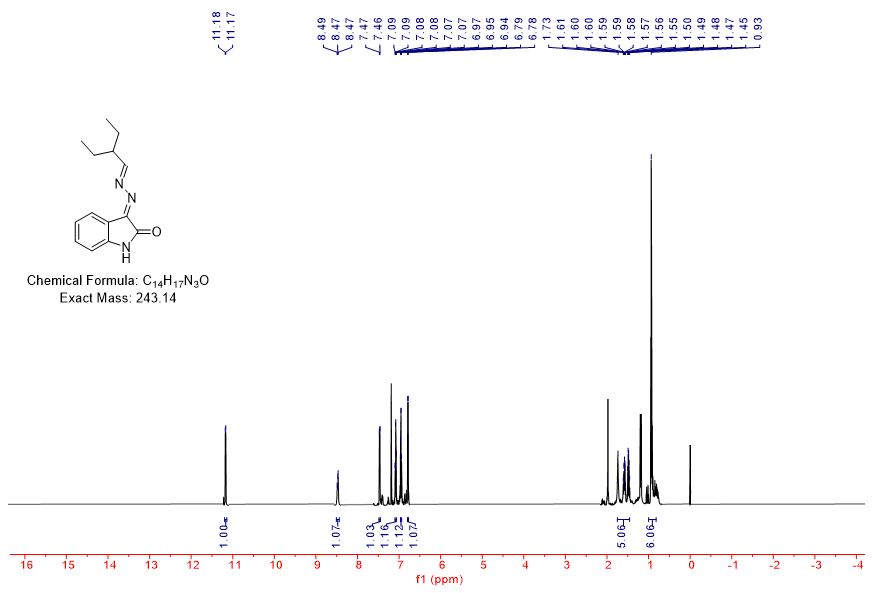


**Figure S12.** ^1^H NMR spectrum of **4b**


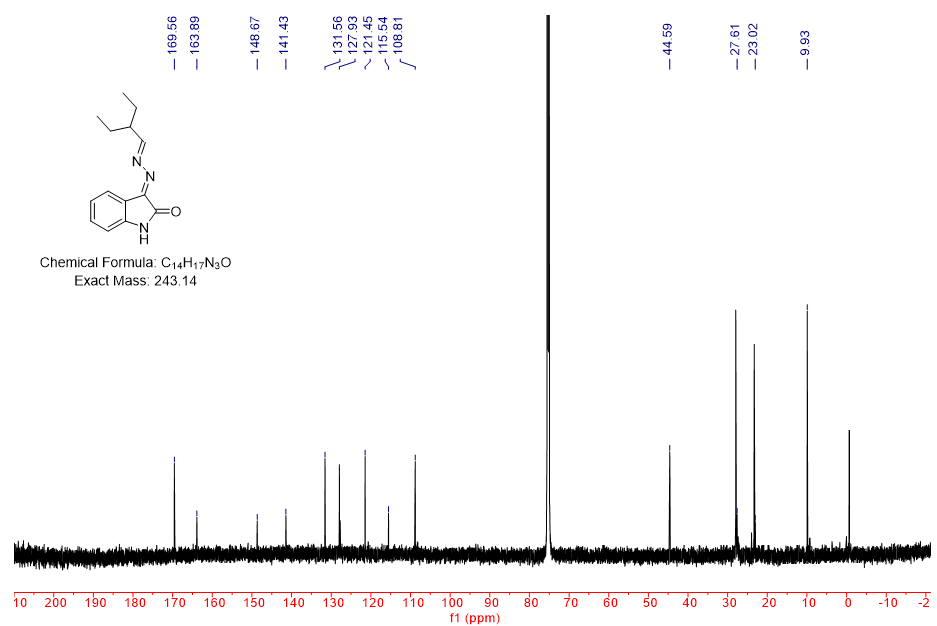


**Figure S13.** ^13^C NMR spectrum of **4b**


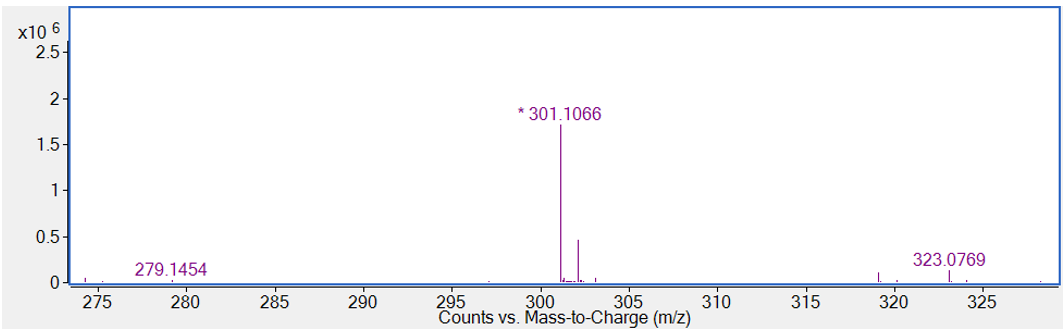


**Figure S14.** HRMS spectrum of **4c**


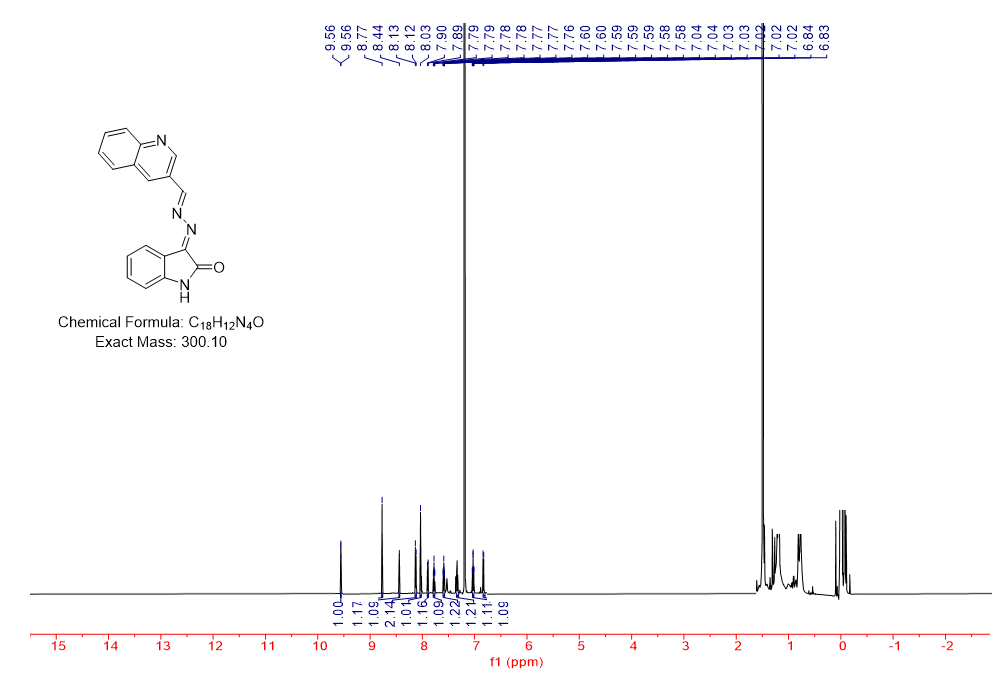


**Figure S15.** ^1^H NMR spectrum of **4c**


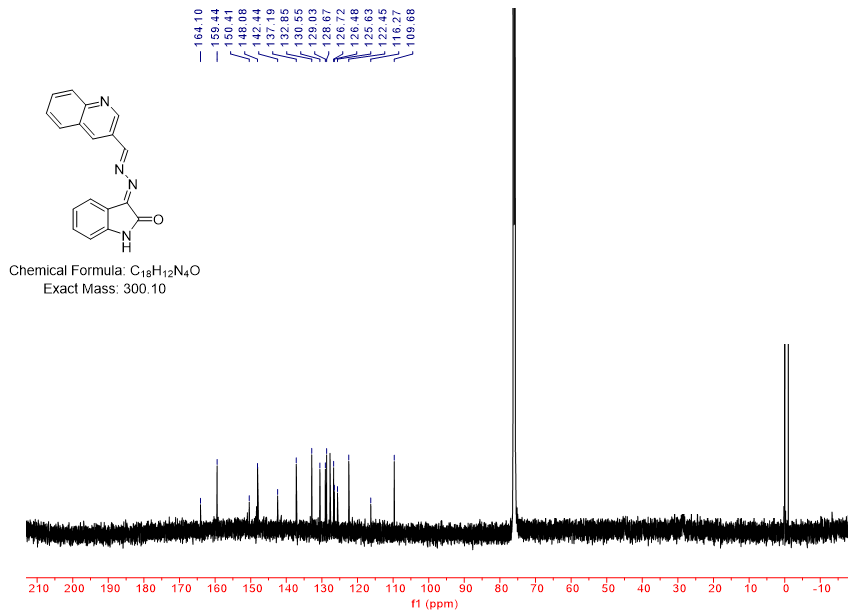


**Figure S16.** ^13^C NMR spectrum of **4c**


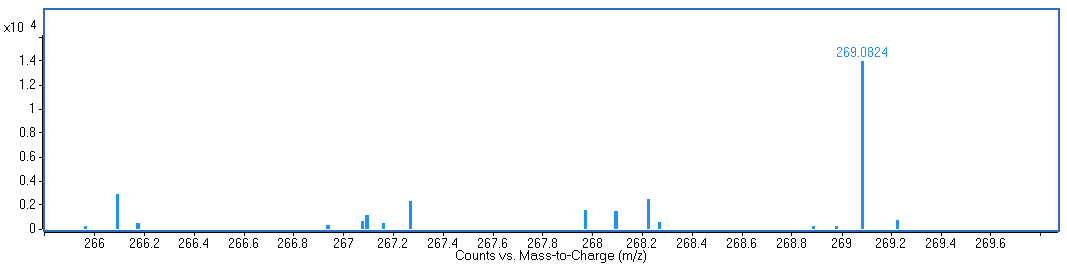


**Figure S17.** HRMS spectrum of **4d**


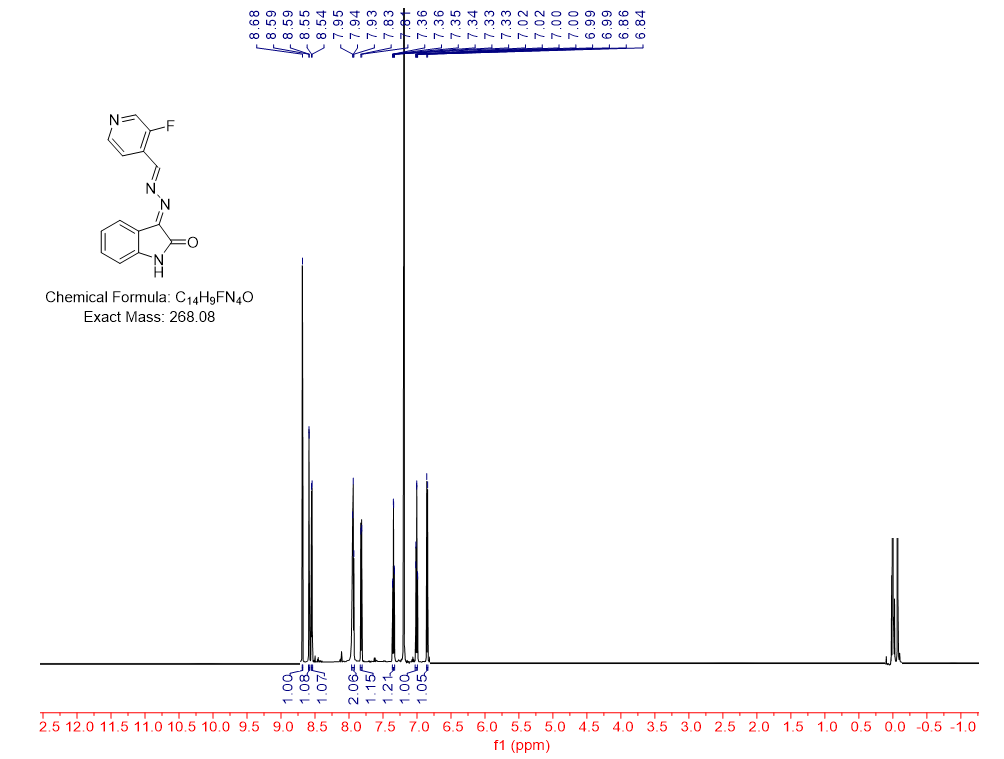


**Figure S18.** ^1^H NMR spectrum of **4d**


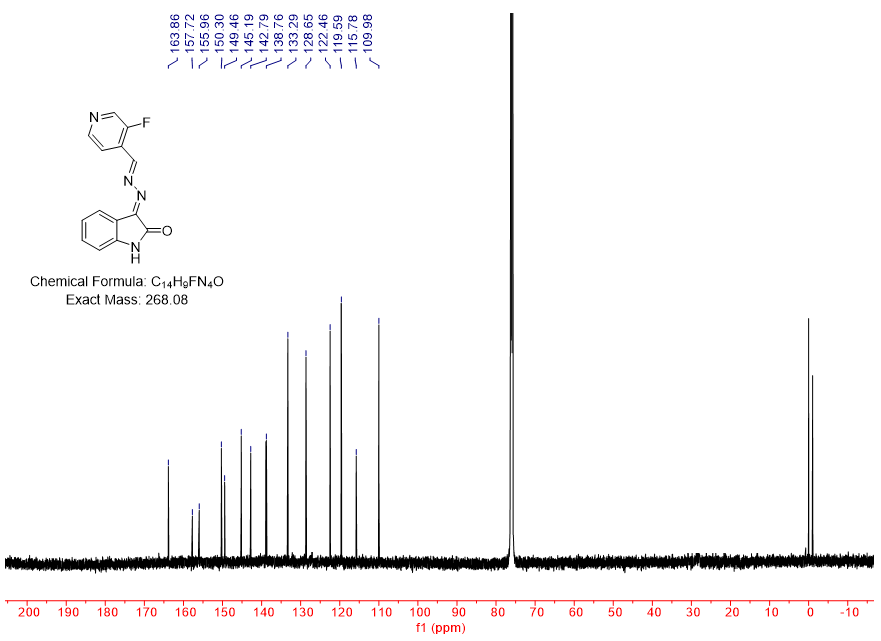


**Figure S19.** ^13^C NMR spectrum of **4d**


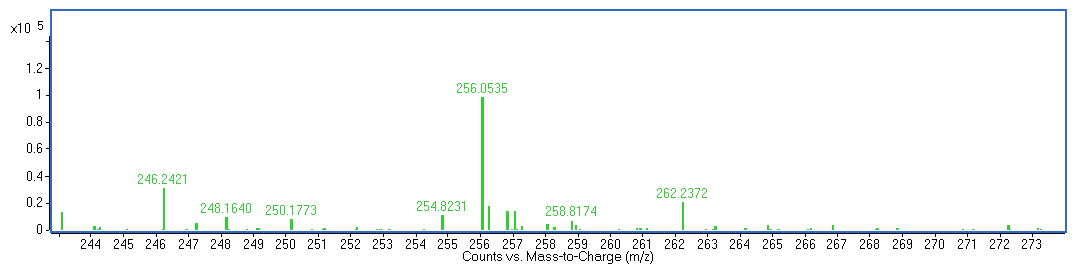


**Figure S20.** HRMS spectrum of **4e**


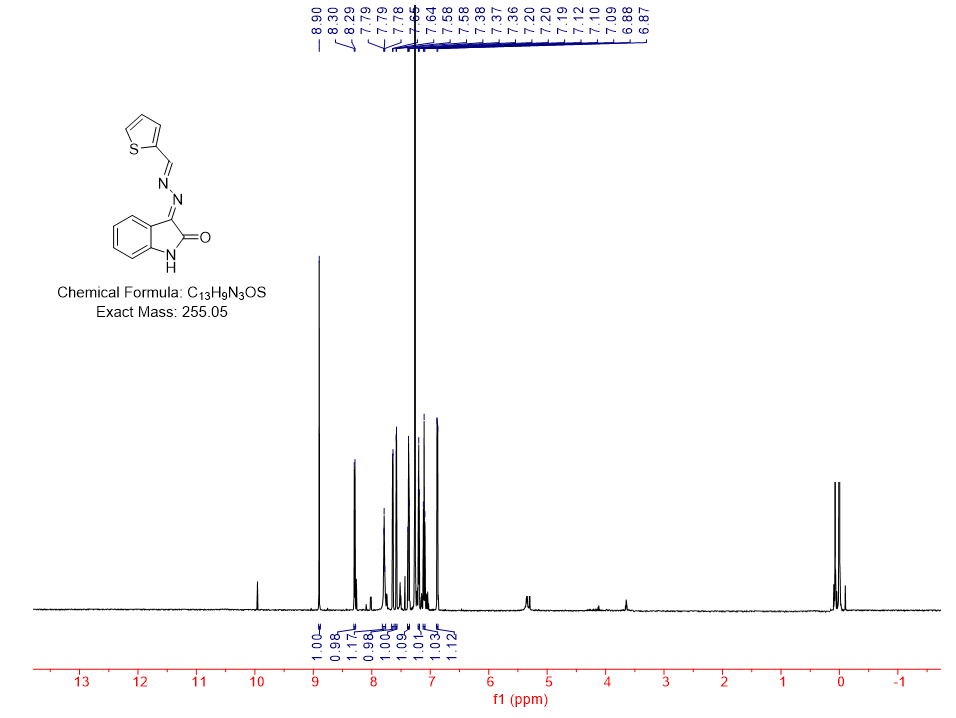


**Figure S21.** ^1^H NMR spectrum of **4e**


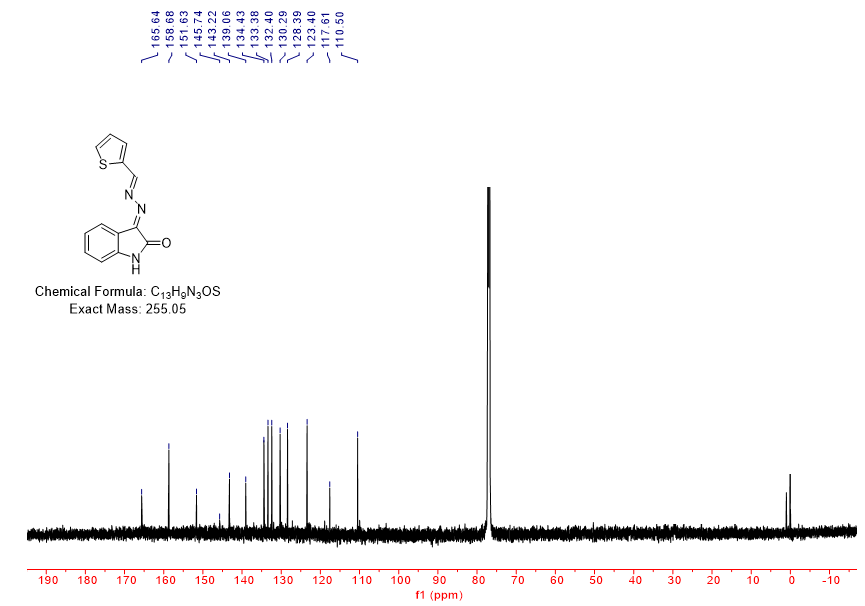


**Figure S22.** ^13^C NMR spectrum of **4e**


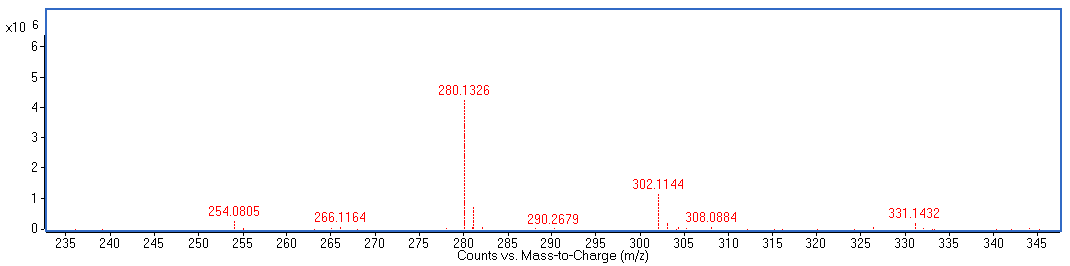


**Figure S23.** HRMS spectrum of **7a**


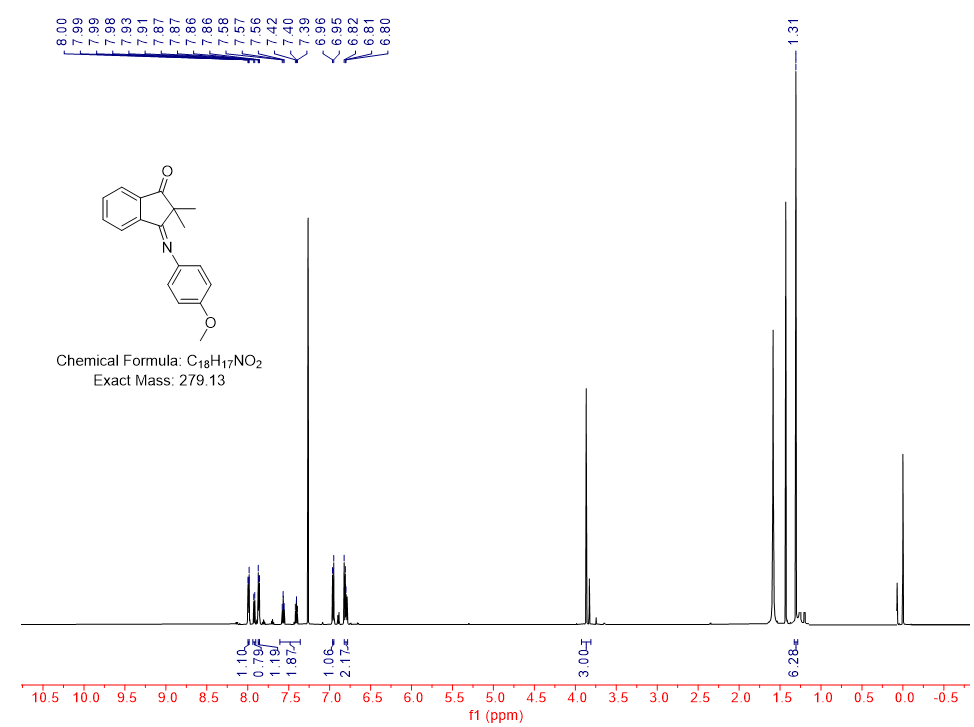


**Figure S24.** ^1^H NMR spectrum of **7a**


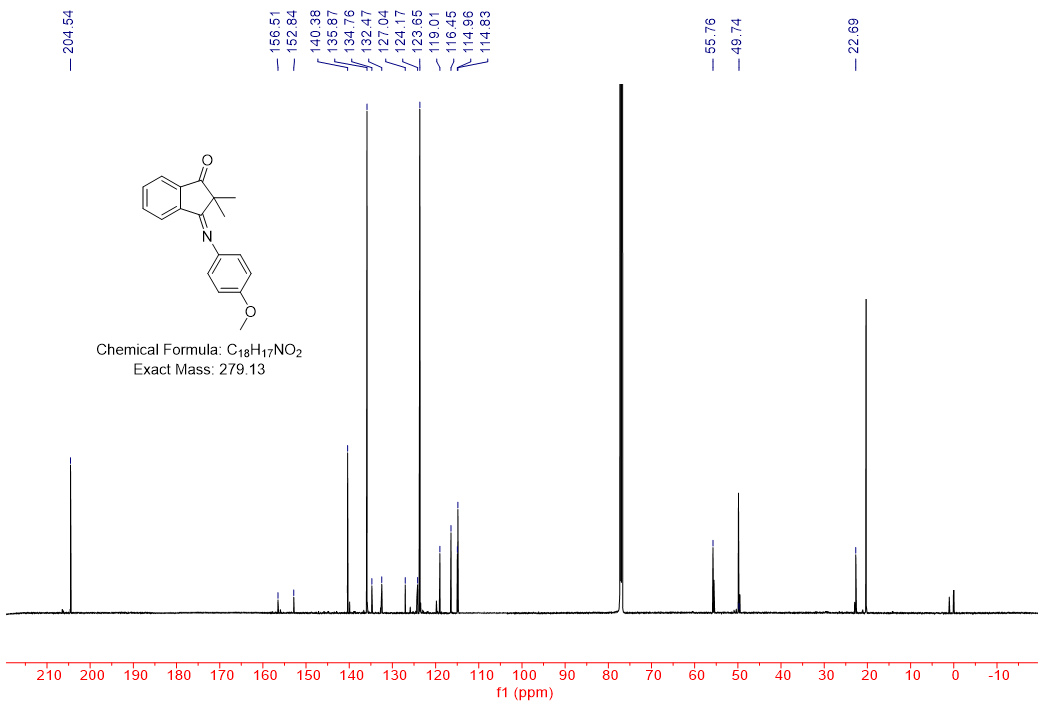


**Figure S25.** ^13^C NMR spectrum of **7a**


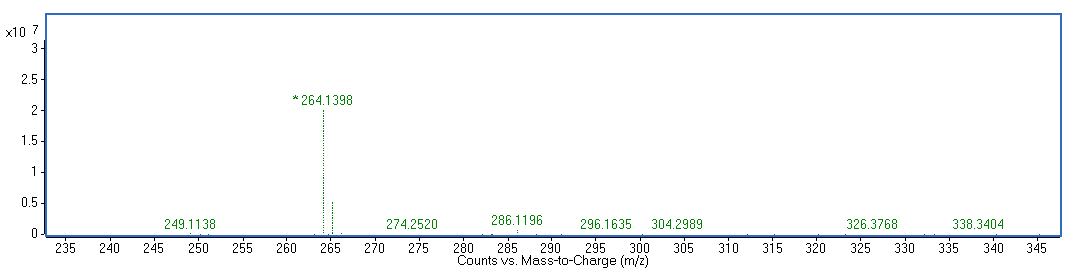


**Figure S26.** HRMS spectrum of **7b**


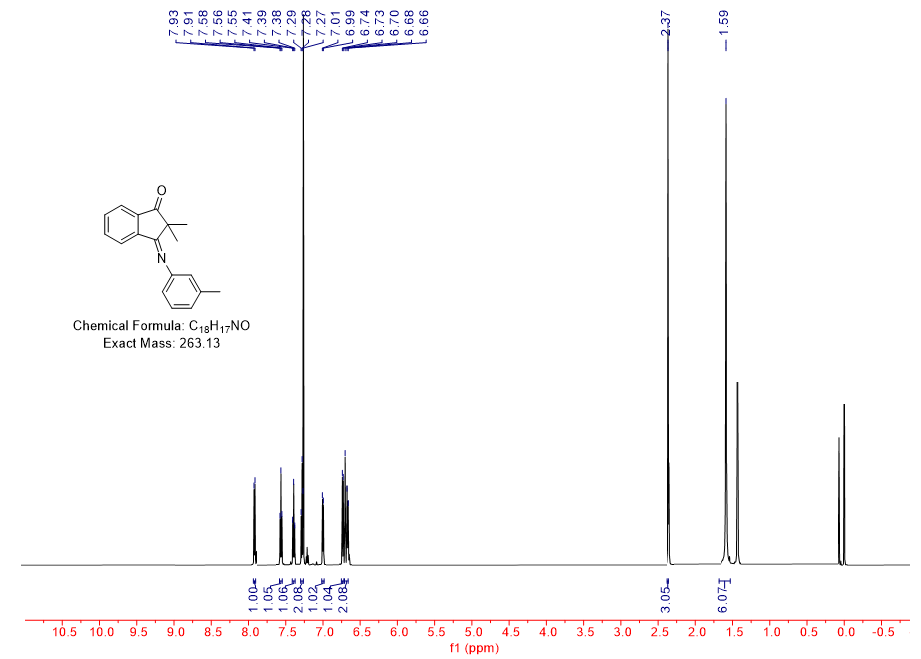


**Figure S27.** ^1^H NMR spectrum of **7b**


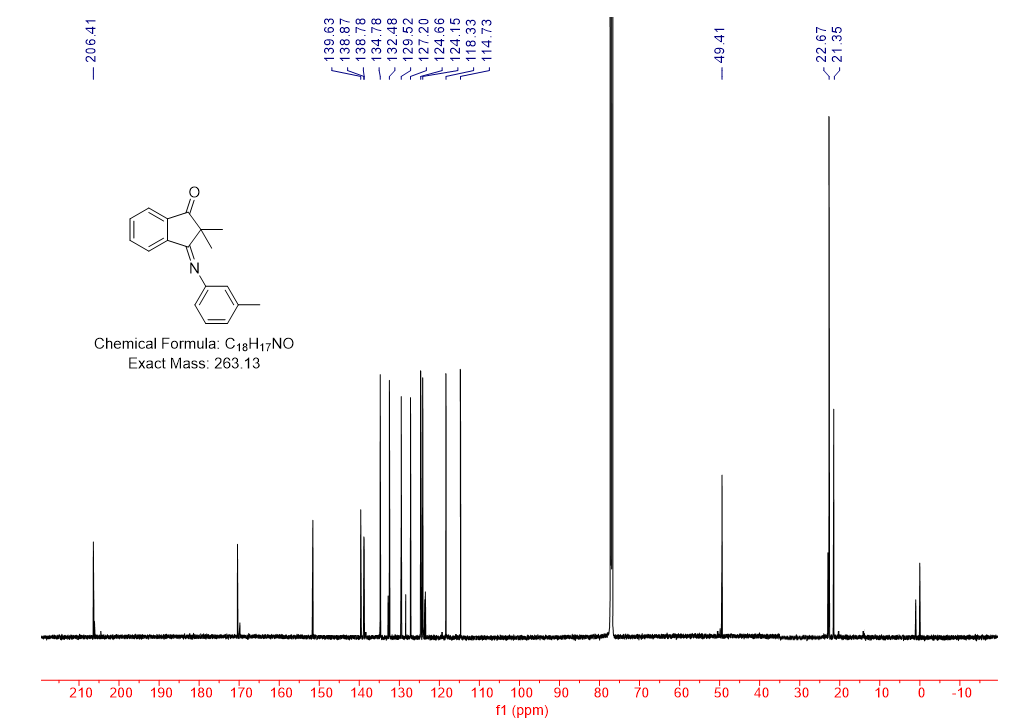


**Figure S28.** ^13^C NMR spectrum of **7b**


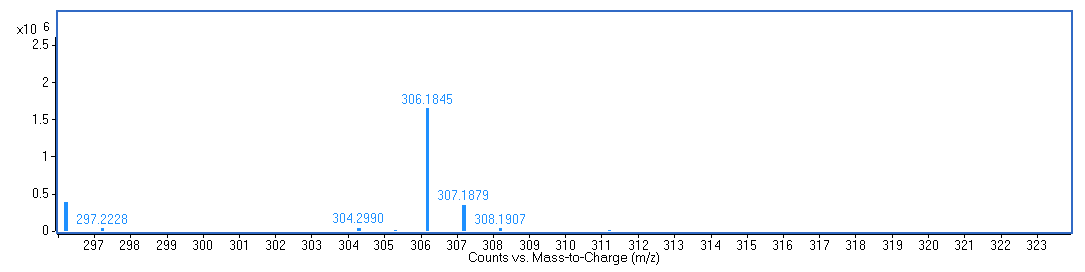


**Figure S29.** HRMS spectrum of **7c**


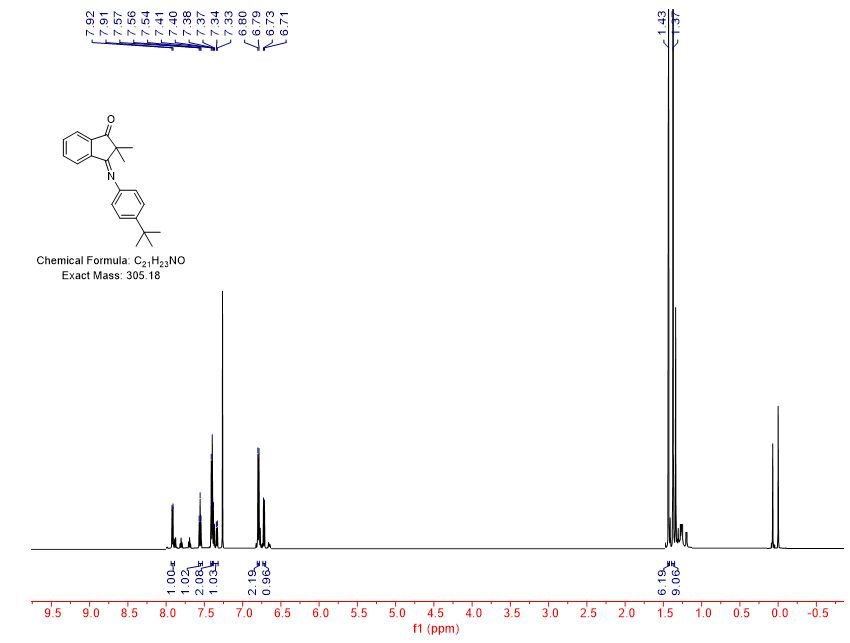


**Figure S30.** ^1^H NMR spectrum of **7c**


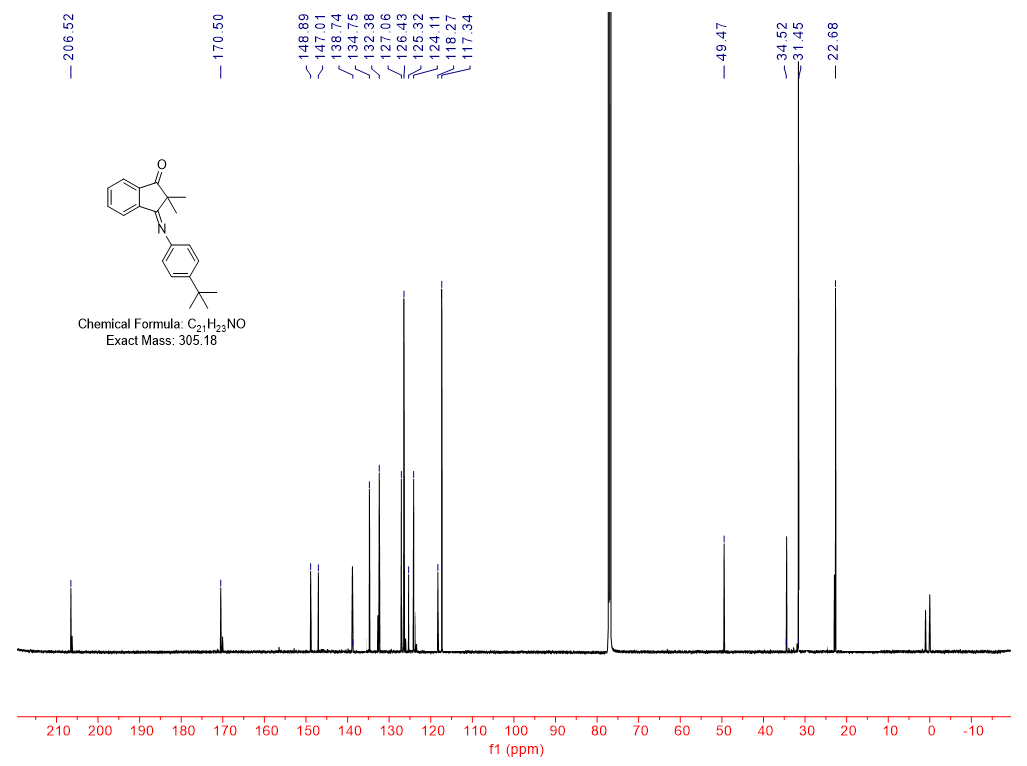


**Figure S31.** ^13^C NMR spectrum of **7c**


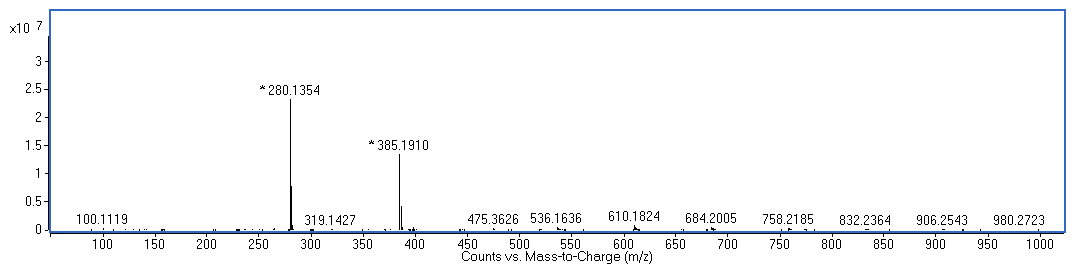


**Figure S32.** HRMS spectrum of **(8a/ZYZ-384)**


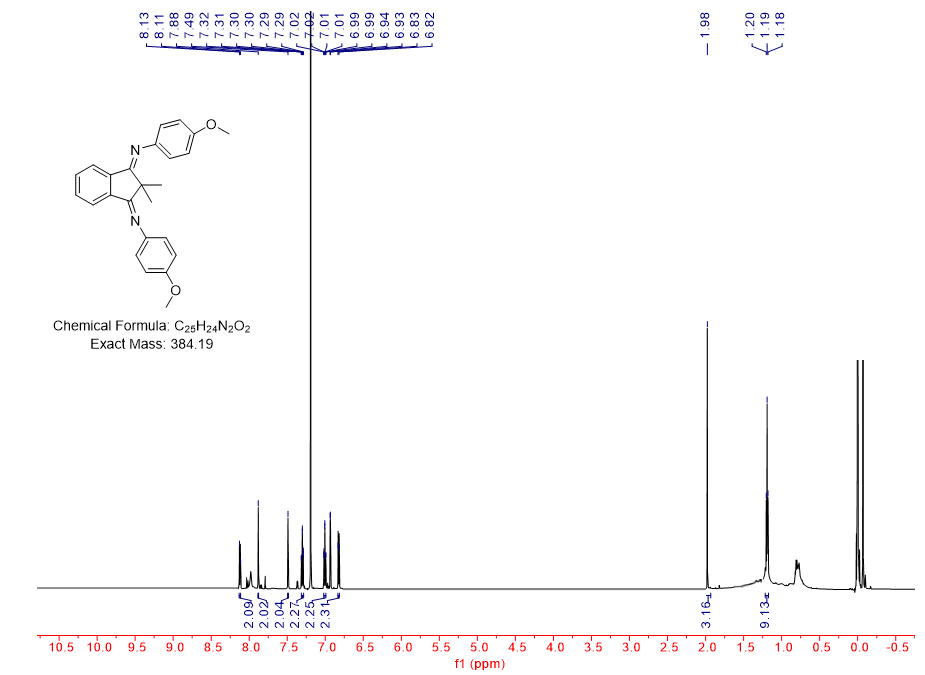


**Figure S33.** ^1^H NMR spectrum of **(8a/ZYZ-384)**


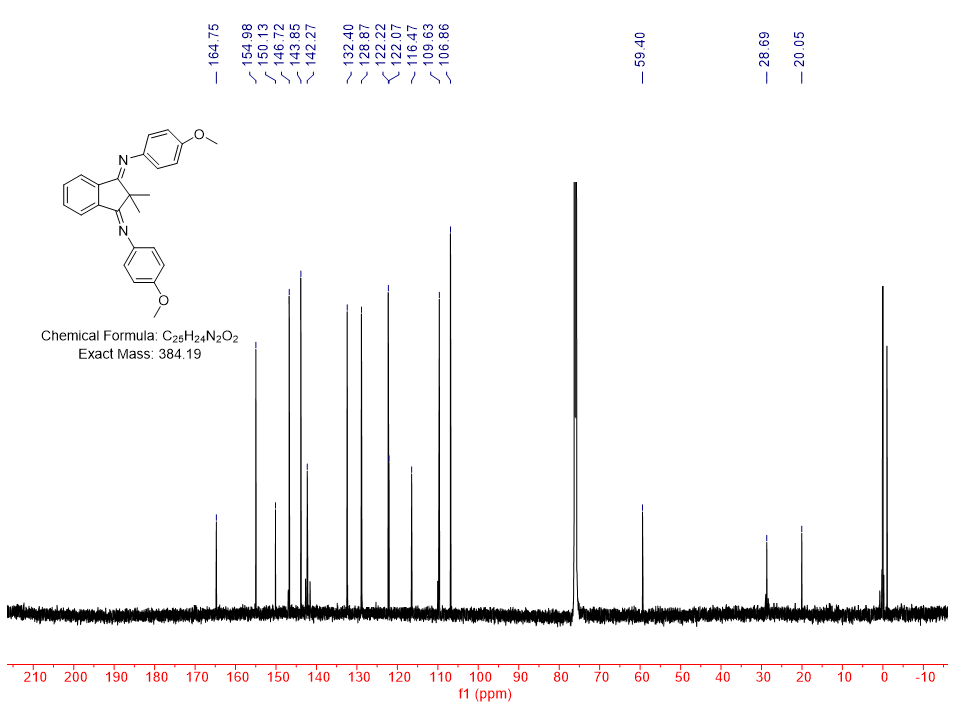


**Figure S34.** ^13^C NMR spectrum of **(8a/ZYZ-384)**


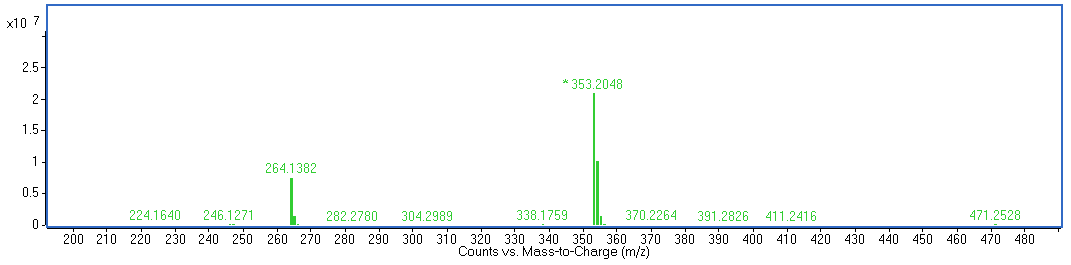


**Figure S35.** HRMS spectrum of **8b**


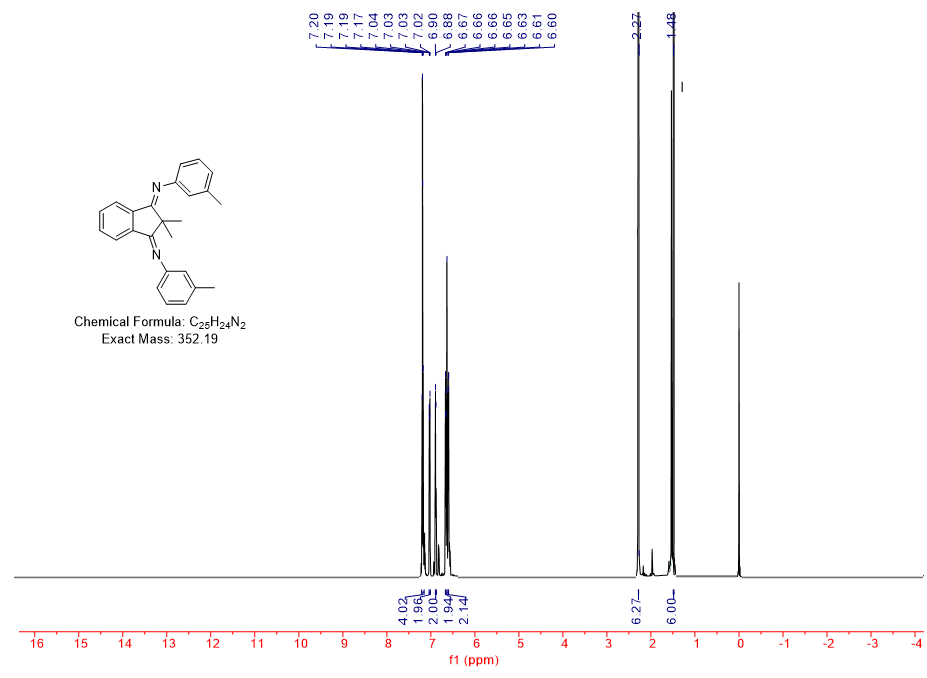


**Figure S36.** ^1^H NMR spectrum of **8b**


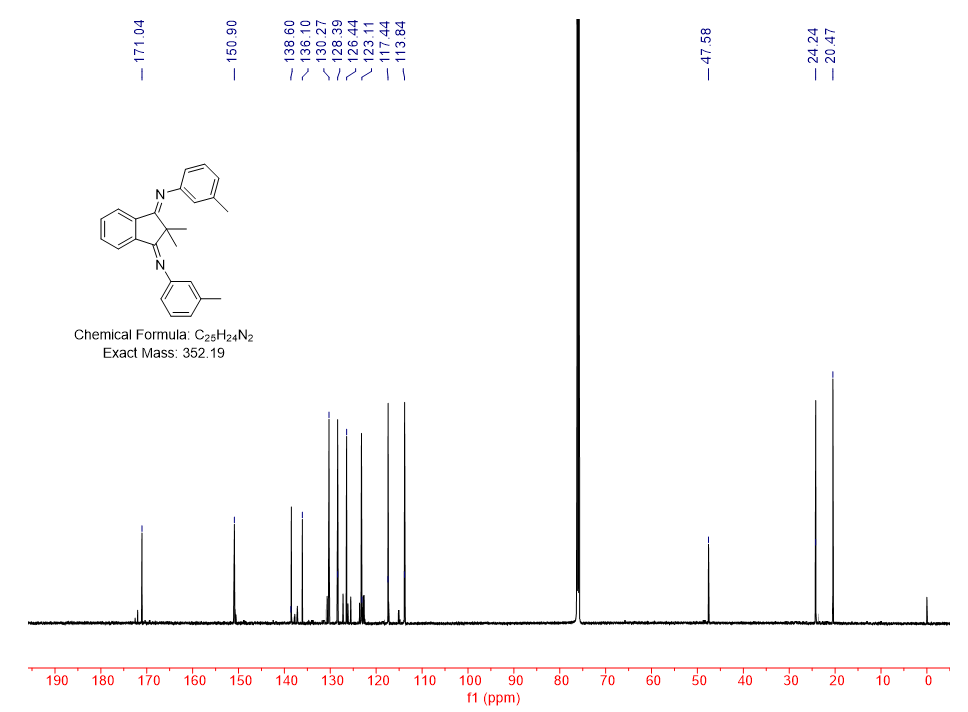


**Figure S37.** ^13^C NMR spectrum of **8b**

1. **Table S2. The gene-specific primer sequences**

| Gene | F/R | Sequences |
| --- | --- | --- |
| actin | F | TCCTCCTGAGCGCAAGTACTCC |
|  | R | CATACTCCTGCTTGCTGATCCAC |
| P21 | F | GCACTTTGATTAGCAGCGGA |
|  | R | GAAAGACAACTACTCCCAGC |
|  | R | GCTCCCCTTTCTTGCGGAGA |
| CyclinD1 | F | CGCGTACCCCGATGCCAAC |
|  | R | CCAGGTGGCGACGATCTTCC |
| CyclinD2 | F | ACACCGACAACTCCATCAAGCC |
|  | R | GACAGCTGCCAGGTTCCACT |
| CDK1 | F | AAACTACAGGTCAAGTGGT |
|  | R | TAAGCACATCCTGAAGACTGA |
| Rac1 | F | TGCCAATGTTATGGTAGATGGAA |
|  | R | TTTTCAAATGATGCAGGACTCAC |
| AKT | F | CAGCATCGCTTCTTTGCCGGTA |
|  | R | CCTGGTGTCAGTCTCCGACGTGA |
| SMYD3 | F | AAAGCTGATGCGATGCTCT |
|  | R | CTGCCAAGAAGTCGAACGGA |
